# Investigations into Chemical Components from *Monascus purpureus* with Photoprotective and Anti-Melanogenic Activities

**DOI:** 10.3390/jof7080619

**Published:** 2021-07-29

**Authors:** Ho-Cheng Wu, Yih-Fung Chen, Ming-Jen Cheng, Ming-Der Wu, Yen-Lin Chen, Hsun-Shuo Chang

**Affiliations:** 1 Graduate Institute of Natural Products, College of Pharmacy, Kaohsiung Medical University, Kaohsiung 807, Taiwan; duncanwu762001@gmail.com (H.-C.W.); yihfungchen@kmu.edu.tw (Y.-F.C.); 2 School of Pharmacy, College of Pharmacy, Kaohsiung Medical University, Kaohsiung 807, Taiwan; 3 Department of Medical Research, Kaohsiung Medical University Hospital, Kaohsiung 807, Taiwan; 4 Bioresource Collection and Research Center (BCRC), Food Industry Research and Development Institute (FIRDI), Hsinchu 300, Taiwan; cmj@firdi.org.tw (M.-J.C.); wmd@firdi.org.tw (M.-D.W.); alc@firdi.org.tw (Y.-L.C.)

**Keywords:** *Monascus purpureus*, xanthonoids, naphthalenone, azaphilone, photoprotection, anti-melanogenesis

## Abstract

*Monascus* species are asexually or sexually reproduced homothallic fungi that can produce a red colorant, specifically the so-called red yeast rice or Anka, which is used as a food ingredient in Asia. Traditional experiences of using *Monascus* for treating indigestion, enhancing blood circulation, and health remedies motivate us to investigate and repurpose *Monascus*-fermented products. Here, two new 5*H*-cyclopenta[*c*]pyridine type azaphilones, 5*S*,6*S*-monaspurpyridine A (**1**) and 5*R*,6*R*-monaspurpyridine A (**2**), two new xanthonoids, monasxanthones A and B (**3** and **4**), one new naphthalenone, monasnaphthalenone (**5**), and one new azaphilone, monapurpurin (**6**), along with two known compounds were isolated from the 70% EtOH extract of a citrinin-free domesticated strain *M. purpureus* BCRC 38110. The phytochemical properties of the xanthonoid and naphthalenone components were first identified from *Monascus* sp. differently from the representative ingredients of polyketide-derived azaphilones. UVB-induced cell viability loss and reactive oxygen species (ROS) overproduction in human keratinocytes were attenuated by monascuspirolide B (**7**) and ergosterol peroxide (**8**), indicating their photoprotective potentials. Ergosterol peroxide (**8**) decreased the melanin contents and tyrosinase activities of mouse melanocytes, depending on the concentration, suggesting their anti-melanogenic effects. In conclusion, six new and two known compounds were isolated from *M. purpureus* BCRC 38110, and two of them exhibited dermal protective activities. The results revealed the novel potential of *M. purpureus* for developing natural cosmeceutics against skin photoaging.

## 1. Introduction

In Asian countries, *Monascus* sp. fermentation products, also called red yeast rice, red koji rice, or Anka, are well-known microbial natural products which have been extensively used for more than 1000 years. The *Monascus*-fermented products have been extensively used for preserving meat and fish, food coloration, and rice wine brewing. Aside from that, the medicinal properties of *Monascus*-fermented products have been described in the ancient Chinese pharmacopeia for easing digestion, smoothing pain, and blood sweeping [1,2]. However, citrinin, a hepato-nephrotoxic mycotoxin, and some toxic components of *Monascus* sp. are the primary concern for the use of *Monascus*-fermented products. Therefore, for safety reasons, screening for the alternative citrinin-free *Monascus* strain is required.

*Monascus* sp. are homothallic fungi and belong to *the* Monascaceae family. It can reproduce sexually (by the formation of cleistothecium with ascospores) and asexually (by the formation of conidia). Either asexual or sexual spores are an effective growth strategy [3,4]. The cleistothecium mycelium of *Monascus* sp. was observed, surrounded with ascospores that were smooth and pigmented. Its ascospores appear to be spherical in shape or slightly ovoid which are easy to observe [4]. Based on the physiological and morphological characteristics, five representative *Monascus* species have been identified, including *M. purpureus*, *M. pilosus*, *M. ruber*, *M. kaoliang*, and *M. anka*. Among them, *M. pilosus*, *M. purpureus* and *M. ruber* have been extensively studied and applied in industrial applications [5].

Usually, *Monascus* sp. produces a group of representive pigments (yellow pigments: monascin and ankaflavin; orange pigments: monascorubrin and rubropunctatin; and red pigments: monascorubramine and rebropunctamine) and monacolins. Interestingly, despite the individual differences among the strains, the culture condition and even the co-cultivations also affect *Monascus* growth and the production of its secondary metabolites [2]. For example, previous studies of *M. purpureus*, one of the representive *Monascus* species, have identified more than one hundred chemical constituents from different strains. Apart from the characteristic red yeast rice secondary metabolites, azaphilones [6,7,8,9,10,11,12,13,14,15,16,17,18,19,20,21,22,23] and monacolins [11,23,24,25,26,27,28,29,30], as well as other types of compounds including alkaloids [7], benzenoids [8,10,13], decalins [31], diterpenes [32], fatty acids [8,33], furanoids [7], isochromane [34], γ-lactam [35], prones [32], sesquiterpenes [32], steroids [8,10,11,32,36,37], siderophore [38], tetralones [32], and triterpenes [32] were also found in *M. purpureus*, indicating the chemical diversity of this species and worthiness of further in-depth investigations.

Many secondary metabolites from red yeast rice products are demonstrated to be active compounds, such as azaphilone pigments, monacolins, dimerumic acid, and γ-aminobutyric acid [16,39,40]. For example, monacolin K is a well-documented metabolite of *Monascus* and used as a cholesterol-lowering reagent via inhibiting 3-hydroxy-3-methylglutaryl coenzyme A (HMG-CoA) reductase, the rate-limiting enzyme for cholesterol biosynthesis [11]. The results from animal studies also suggested the in vivo hypolipidemic effects of monacolins via decreasing the triglycerides, total cholesterol, and low-density lipoproteins and increasing high-density lipoproteins. The azaphilone pigments in *Monascus* sp. were demonstrated to have a range of biological activities, such as anti-inflammatory [41,42,43], anti-diabetic [44], antimicrobial [45,46], and anti-obesity [47] activities. The secondary metabolites, especially pigments and monacolins from *Monascus* sp., were identified as anti-tumor and cytotoxic components against lung cancer [48], colon cancer [28], prostate cancer [49], liver cancer [50], prostate cancer [49], and breast cancer [51]. Additionally, the orange pigment monascorubrin and yellow pigment monascin suppressed the tumorigenesis in mouse skin carcinogenesis tests [52,53], motivating us to further investigate the skin-protective activities of *Monascus*.

Through our long-term research on *M. purpureus*, *M. purpureus* BCRC 38110, a particular citrinin-free strain, was considered a valuable strain for further investigation. Our previous studies of *M. purpureus* BCRC 38110 revealed some bioactive compounds with anti-inflammatory activities [54,55] in lipopolysaccharide (LPS)-stimulated RAW 264.7 macrophages. With bioactivity-guided fractionation of *M. purpureus* BCRC 38110, two unpresented 5′,6′-dihydrospiro[isochromane-1,2′-pyran]-4′(3′*H*)-one derivatives were also found [55]. These promising results motivated us to investigate additional compounds with novel structures and potent bioactivities from *M. purpureus* BCRC 38110.

The skin is the largest organ of our body, forming the first line of defense against the detrimental effects of environmental and xenobiotic agents to protect the body’s internal organs. The epidermis is the outermost of the three layers (i.e., the epidermis, dermis, and hypodermis) that make up the skin. Keratinocytes, the main epidermal cell, undergo differentiation to form distinct layers of the epidermis. Among various environmental stimuli, ultraviolet rays (UVRs) are the most common causative factor for inducing skin photodamage. Excessive UVB exposure usually causes skin photoaging, inflammation, and even photocarcinogenesis. UV radiation-induced reactive oxygen species (ROS), a major contributing factor to photodamage, are known to cause the strained cellular antioxidant defense system and activate pro-inflammatory responses in human keratinocytes [56,57]. Keratinocyte cell models are critical for studying the biology, photodamage, and photoprotection of skin. Here, in an attempt to explore the potentials of *Monascus* sp. for protecting the skin from photodamage, the study model of UVB-irradiated human keratinocyte HaCaT cells was applied to evaluate the cytoprotective and antioxidant properties of compounds from *M. purpureus* BCRC 38110.

In humans, melanocytes synthesize melanin for protecting the skin against the carcinogenic and deleterious effects of UV radiation [58]. However, the excessive production of melanin affects the appearance of the skin and may increase the risk of melanoma. Upon UV exposure, keratinocytes secrete an α-melanocyte-stimulating hormone (α-MSH) which binds to the melanocortin receptors on melanocytes and stimulates melanin biosynthesis [59,60]. Tyrosinase, the key rate-limiting enzyme for the biosynthesis of melanin pigment in the melanocytes, catalyzes the conversion of l-tyrosine to l-3,4-dihydroxyphenylalanine (l-DOPA) and *O*-dopaquinone via hydroxylation and oxidation reactions, respectively [57,60,61]. The subsequent metabolism of dopaquinone leads to the formation of melanin. Thus, reducing the melanin synthesis and inhibiting tyrosinase or tyrosinase-like enzymes is a dominant strategy for skin lightening and hyperpigmentation control [62]. This study investigated the anti-melanogenic potentials of secondary metabolites derived from *M. purpureus* BCRC 38110 with the study model of α-MSH-simulated mouse melanoma B16-F10 cells.

## 2. Materials and Methods

### 2.1. General Experimental Procedures

Optical rotations were taken using a Jasco P-2000 polarimeter (Jasco, Kyoto, Japan). The IR spectra (ATR) were obtained with an FT/IR-6000 FTIR spectrometer (Jasco, Kyoto, Japan). The UV spectra were determined on a Hitachi-U5100 ratio beam spectrophotometer (Hitachi, Tokyo, Japan). The circular dichroism spectra were recorded on a JASCO J-810 CD spectrometer. High-resolution electrospray ionization-mass spectrometry (HRESI-MS) were obtained on a Bruker APEX II mass spectrometer (Bruker, Karlsruhe, Germany). The NMR spectra were run on a Varian Mercuryplus-400/VNMRS-600 spectrometer (Varian, Inc. Vacuum Technologies, Ave, Lexington, MA, USA). Column chromatography was carried out with glass columns packed with silica gel (LiChroprep^®^ Si 60, 25–40 mesh; Merck, Darmstadt, Germany) and RP-18 (LiChroprep^®^ RP-18, 15–25 mesh; Merck, Darmstadt, Germany). Medium-performance liquid chromatography (MPLC) was performed using a VSP-3050 EYELA ceramic pump (EYELA, Kyoto, Japan). Further purification steps were performed by high-performance liquid chromatography (HPLC) using a JASCO HPLC LC-4000 (JASCO Corporation, Tokyo, Japan) with a photodiode array detector.

### 2.2. Cultivation and Preparation of Red Yeast Rice

*Monascus purpureus* BCRC 38110 was supplied by the Bioresource Collection and Research Center (BCRC) of the Food Industry Research and Development Institute (FIRDI) in Taiwan and maintained on the potato dextrose agar (PDA; Difco, Detroit, MI, USA). The culture was maintained and sporulated on PDA slants (25 °C, 14 days). The spores were harvested with sterile water and seeded (5 × 10^5^) into 300-mL shake flasks containing 50 mL RGY medium (3% rice starch, 7% glycerol, 1.1% polypeptone, 3.2% soybean powder, 0.2% MgSO_4_, and 0.2% NaNO_3_). The flasks were incubated on a rotary shaker at 150 rpm and 25 °C for three days. The enriched mycelium (100 mL) was mixed with a 100 mL RGY medium and then inoculated into plastic boxes (25 × 30 cm) containing 2 kg sterile rice at 25 °C. A 150-mL RGY medium was added for maintaining the growth of yeasts on day 7. After 28 days of cultivation, the red yeast rice was harvested and lyophilized for the extraction of metabolites.

### 2.3. Extraction and Isolation

The solid-state fermentation of *Monascus purpureus* (1.5 kg) was extracted with 70% EtOH three times at room temperature. The ethanolic syrup extract was partitioned with EtOAc and H_2_O (1:1) to give an EtOAc-soluble fraction (15.6 g) and a H_2_O-soluble fraction. The EtOAc-soluble fraction was subjected to column chromatography (Silica gel; *n*-hexane/EtOAc 100:1 to 100% acetone, then washed with 100% methanol) to produce 15 subfractions (Frs. 1-15). Fr. 8 was chromatographed with *n*-hexane/EtOAc 3:1 to give 12 fractions. Fr. 8-9 was subjected to MPLC (silica gel; dichloromethane/acetone 20:1) to obtain 14 subfractions, which contained **8** (2.3 mg). Fr. 12 was subjected to MPLC (silica gel; *n*-hexane/acetone 3:1) to obtain 14 fractions. Fr. 12-10 through 12-12 were subjected to MPLC (silica gel; dichloromethane/EtOAc 4:1) to give **3** (1.1 mg). Fr. 14 was subjected to MPLC (silica gel; dichloromethane/EtOAc 3:2) to give eleven subfractions. Fr. 14-4 through 14-7 were combined and chromatographed with a C-18 column eluted by a water/acetone mixture (1:1) to obtain **7** (43.0 mg). Fr. 15 (1.2 g) was subjected to MPLC (RP-18; H_2_O/methanol 1:1.5) to give nineteen subfractions. Fr. 15-3 was purified by semi-prepared HPLC on a diphenyl column (H_2_O/methanol 3:2, flow = 2 mL/min) to give **1** (1.0 mg) and **2** (0.7 mg). Fr. 15-3-3 was purified by semi-prepared HPLC on a diphenyl column (H_2_O/methanol 3:2, flow = 2 mL/min) to give **5** (1.8 mg). Fr. 15-11 was purified by silica gel with dichloromethane/acetone 4:1 to give **6** (1.0 mg). Compound **4** (2.2 mg) was yielded by semi-prepared HPLC on an RP-18 silica gel (H_2_O/methanol 1:2, flow = 2 mL/min) from Fr. 15-11-3.

### 2.4. Spectral Data

#### 2.4.1. 5*S*,6*S*-Monaspurpyridine A (**1**)

Yellowish syrup. ${\left[\alpha \right]}_{D}^{\mathrm{26}}$: −10.3 (*c* 0.05, MeOH). UV (MeOH) λ_max_ (log *ε*) 204 (4.17), 250 sh (4.84), 274 sh (3.55) nm. IR *ν*_max_ (ATR) 3437 (OH), 1737 (C=O), 1607 (C=C) cm^−1^. CD (MeOH) λ_ext_ 201 (Δε +7.5), 227 (Δε −10.2), 247 (Δε +8.2), 347 (Δε −1.8) nm. HRESIMS *m/z*: 332.11026 [M + Na]^+^, C_15_H_19_NO_6_Na, Calcd.: C_15_H_19_NO_6_Na, 332.11046. ^1^H NMR (CD_3_OD, 600 MHz) *δ*: 1.07 (3H, t, *J* = 6.0 Hz, H-14), 1.24 (3H, d, *J* = 6.0 Hz, H-10), 1.28 (3H, s, H-11), 3.00 (2H, m, H-8), 4.07 (2H, m, H-13), 4.22 (1H, sextet, *J* = 6.0 Hz, H-9), 7.58 (1H, s, H-4), 8.87 (1H, s, H-1). ^13^C NMR (CD_3_OD, 150 MHz) *δ*: 14.1 (C-14), 21.8 (C-11), 23.5 (C-10), 48.6 (C-8), 63.5 (C-13), 68.4 (C-9), 84.8 (C-5), 87.1 (C-6), 120.8 (C-4), 129.8 (C-7a), 145.6 (C-1), 159.5 (C-4a), 167.6 (C-3), 171.8 (C-12), 203.5 (C-7).

#### 2.4.2. 5*R*,6*R*-Monaspurpyridine A (**2**)

Yellowish syrup. ${\left[\alpha \right]}_{D}^{\mathrm{26}}$: +40.6 (*c* 0.04, MeOH). UV (MeOH) λ_max_ (log *ε*) 204 (4.18), 252 sh (3.82), 272 sh (3.46) nm. IR *ν*_max_ (ATR) 3375 (OH), 1728 (C=O), 1604 (C=C) cm^−1^. CD (MeOH) λ_ext_ 203 (Δε +0.4), 215 (Δε +8.8), 228 (Δε +9.0), 248 (Δε −6.5), 343 (Δε +2.2), 396 (Δε −0.6) nm. HRESIMS *m/z*: 332.11020 [M + Na]^+^, C_15_H_19_NO_6_Na, Calcd.: C_15_H_19_NO_6_Na, 332.11046. ^1^H NMR (CD_3_OD, 600 MHz) *δ*: 1.07 (3H, t, *J* = 6.0 Hz, H-14), 1.24 (3H, d, *J* = 6.0 Hz, H-10), 1.28 (3H, s, H-11), 3.00 (2H, m, H-8), 4.07 (2H, m, H-13), 4.22 (1H, sextet, *J* = 6.0 Hz, H-9), 7.58 (1H, s, H-4), 8.87 (1H, s, H-1). ^13^C NMR (CD_3_OD, 150 MHz) *δ*: 14.1 (C-14), 21.8 (C-11), 23.5 (C-10), 48.6 (C-8), 63.5 (C-13), 68.4 (C-9), 84.8 (C-5), 87.1 (C-6), 120.8 (C-4), 129.8 (C-7a), 145.6 (C-1), 159.5 (C-4a), 167.6 (C-3), 171.8 (C-12), 203.5 (C-7).

#### 2.4.3. Monasxanthone A (**3**)

Yellowish solid. ${\left[\alpha \right]}_{D}^{\mathrm{26}}$: −97.3 (*c* 0.045, CHCl_3_). UV (MeOH) λ_max_ (log *ε*) 254 (4.04), 278 sh (4.02), 360 (3.96) nm. UV (MeOH + KOH) λ_max_ (log *ε*) 254 sh (3.95), 332 (3.96) nm. IR *ν*_max_ (ATR) 3437 (OH), 1713 (C=O), 1672 (C=O), 1620, 1572, 1486 (aromatic ring) cm^−1^. HRESIMS *m/z*: 409.12561 [M + Na]^+^, C_21_H_22_O_7_Na, Calcd.: C_21_H_22_O_7_Na, 409.12577. For ^1^H NMR (CDCl_3_, 600 MHz) and ^13^C NMR (CDCl_3_, 150 MHz) data, see Table 1.

#### 2.4.4. Monasxanthone B (**4**)

Yellowish solid. ${\left[\alpha \right]}_{D}^{\mathrm{26}}$: +55.9 (*c* 0.072, CHCl_3_). UV (MeOH) λ_max_ (log *ε*) 270 (4.27), 360 (4.12) nm. UV (MeOH + KOH) λ_max_ (log *ε*) 268 (4.14), 330 (4.18), 430 (4.10) nm. IR *ν*_max_ (ATR) 3429 (OH), 1710 (C=O), 1674 (C=O), 1620, 1573 (aromatic ring) cm^−1^. HRESIMS *m/z*:425.12047 [M + Na]^+^, C_21_H_22_O_8_Na, Calcd.: C_21_H_22_O_8_Na, 425.12069. For ^1^H NMR (CDCl_3_, 600 MHz) and ^13^C NMR (CDCl_3_, 150 MHz) data, see Table 1.

#### 2.4.5. Monasnaphthalenone (**5**)

Yellowish solid. ${\left[\alpha \right]}_{D}^{\mathrm{26}}$: ±0 (*c* 0.09, MeOH). IR *ν*_max_ (ATR) 3422 (OH), 1707, 1674 (C=O), 1620, 1576 (aromatic ring) cm^−1^. HRESIMS *m/z*: 355.11392 [M + Na]^+^, C_18_H_20_O_6_Na, Calcd.: C_18_H_20_O_6_Na, 355.11576. ^1^H NMR (acetone-*d*_6_, 700 MHz) *δ*: 1.18 (3H, d, *J* = 4.8 Hz, H-16), 1.60 (3H, s, H-11), 2.48 (3H, s, H-10), 2.67 (1H, dd, *J* = 13.5, 3.9 Hz, H-14b), 2.74 (1H, dd, *J* = 13.5, 6.9 Hz, H-14a), 3.91 (1H, d, *J* = 15.0 Hz, H-12b), 4.10 (1H, d, *J* = 15.0 Hz, H-12a), 4.24 (1H, m, H-15), 5.98 (1H, s, H-6), 6.87 (1H, d, *J* = 7.2 Hz, H-3), 7.37 (1H, d, *J* = 7.2 Hz, H-4). ^13^C NMR (acetone-*d*_6_, 175 MHz) *δ*: 24.6 (C-16), 32.7 (C-11), 33.4 (C-10), 49.8 (C-12), 52.8 (C-14), 65.4 (C-15), 79.3 (C-8), 115.7 (C-3), 122.9 (C-6), 123.2 (C-4a), 129.7 (C-4), 131.9 (C-1), 144.7 (C-8a), 152.2 (C-5), 156.9 (C-2), 204.5 (C-10), 204.6 (C-7), 206.8 (C-13).

#### 2.4.6. Monapurpurin (**6**)

Yellowish solid. ${\left[\alpha \right]}_{D}^{\mathrm{26}}$: +27.4 (*c* 0.05, MeOH). UV (MeOH) λ_max_ (log *ε*) 210 (4.36), 236 sh (4.08), 290 (3.62) nm. IR *ν*_max_ (ATR) 3399 (OH), 1739 (C=O), 1627, 1560, 1540 (aromatic ring) cm^−1^. HRESIMS *m/z*: 297.07357 [M + Na]^+^, C_15_H_14_O_5_Na, Calcd.: C_15_H_14_O_5_Na, 297.07334. ^1^H NMR (acetone-*d*_6_, 600 MHz) *δ*: 1.26 (3H, d, *J* = 6.0 Hz, H-13), 1.68 (3H, d, *J* = 6.6 Hz, H-10), 2.65 (1H, dd, *J* = 14.0, 7.8 Hz, H-11b), 2.70 (1H, dd, *J* = 14.0, 5.1 Hz, H-11a), 3.97 (1H, d, *J* = 6.6 Hz, OH-12), 4.21 (1H, m, H-12), 5.78, (1H, q, *J* = 6.6 Hz, H-1), 6.69, (1H, s, H-8), 7.78 (1H, s, H-9), 8.51 (1H, s, H-4). ^13^C NMR (acetone-*d*_6_, 150 MHz) *δ*: 21.1 (C-10), 24.4 (C-13), 45.0 (C-11), 66.2 (C-12), 79.2 (C-1), 106.1 (C-8), 120.6 (C-9), 122.7 (C-4a), 126.7 (C-3a), 128.4 (C-4), 143.9 (C-8a), 157.9 (C-9a), 160.3 (C-7), 162.8 (C-5), 169.7 (C-3).

### 2.5. Bioactivity Assays

#### 2.5.1. Cell Culture

Mouse melanoma B16-F10 cells (BCRC 60031) were purchased from BCRC in Taiwan. A human keratinocyte HaCaT line (Cellosaurus accession number: CVCL_0038) is a spontaneously transformed human keratinocyte line established from the histologically normal skin of a 62-year-old adult [63], which maintains a full epidermal differentiation capacity similar to normal keratinocytes and remains non-tumorigenic. The HaCaT cells were kindly provided by Professor Tsung-Lin Cheng in the Department of Physiology from the College of Medicine at Kaohsiung Medical University in Kaohsiung, Taiwan [64]. The B16-F10 and HaCaT cells were cultured in Dulbecco’s Modified Eagle Medium (DMEM) supplemented with 4.5g/L glucose, 2 mM G-glutamine, 100 U/mL penicillin, 100 μg/mL streptomycin, 0.25 μg/mL amphotericin B, and 10% fetal bovine serum (Sigma-Aldrich, St. Louis, MO, USA), and they were maintained at 37 °C in a humidified incubator with 5% CO_2_ and 95% air. The cell culture medium was replaced every 2–3 days, and confluent cells were passed in a split ratio of 1:3 to 1:5 every 3–5 days with trypsinization.

#### 2.5.2. Cell Viability Assay

The cell viability of the HaCaT and B16-F10 cells was determined with alamarBlue^®^ (resazurin) fluorescent dye. Briefly, the cells were plated onto 96-well plates for 24 h, and the treatments were applied with the indicated concentrations and incubation times. After treatment, 10 μL of alamarBlue^®^ Cell Viability Reagent (Thermo Fisher Scientific, Waltham, MA, USA) was added into the culture medium, and then the plates were incubated for 2 h at 37 °C in a cell culture incubator. Fluorescence with an excitation wavelength of 560 nm and emission of 590 nm, recorded using a fluorescent microplate reader (Bio-Tek Synergy HT, Winooski, VT, USA), which was used to calculate the cell viability.

#### 2.5.3. Determination of Anti-Melanogenic Potentials in Mouse Melanoma B16-F10 Cells

Alpha-MSH-stimulated tyrosinase activities in B16-F10 cells were used as an in vitro model for evaluating the anti-melanogenic potentials of bioactive products [57,60,65]. Briefly, B16-F10 cells were treated with 50 nM α-MSH for 24 h and then treated with vehicle or testing compounds for 48 h. The cells were collected with trypsinization and centrifuged at 12,000 rpm and 4 °C for 10 min. The cellular melanin content and tyrosinase activity were determined as described previously [65,66].

Briefly, for determining the melanin content, the cell pellets were then suspended in 2.0 N NaOH and incubated at 95 °C for 15 min. The absorbance at 405 nm was measured using a microplate reader (Bio-Tek Synergy HT, Winooski, VT, USA). The cellular melanin content was calculated as follows: Melanin content (%) = (absorbance of the tested cells/absorbance of the basal control cells) × 100.

Briefly, for determining the tyrosinase activity, the cell pellets were lysed with 150 µL PBS containing 1% Triton X-100 and 0.1 mM phenylmethylsulfonyl fluoride prior to centrifugation. The supernatants were mixed with L-DOPA (100 µL, 1 mg/mL, dissolved in PBS; Sigma-Aldrich, St. Louis, MO, USA) for 3 h at 37 °C. The absorbance at 490 nm was measured using a microplate reader (Bio-Tek Synergy HT, Winooski, VT, USA). The tyrosinase activity was calculated as follows: tyrosinase activity (%) = (absorbance of the tested cells/absorbance of the basal control cells) × 100.

#### 2.5.4. UV Irradiation in Human Keratinocyte HaCaT Cells

The HaCaT cell line has been a widely used keratinocyte monolayer culture model for investigating photodamage, photoprotection, and therapeutic interventions for skin diseases [67,68]. The HaCaT cells were pretreated with vehicle or testing compounds at 37 °C for 6 h, washed with PBS, and then incubated with PBS for UVB irradiation. Immediately after that, the cells were exposed to UVB irradiation (40 mJ/cm^2^) using a CL-1000M UV crosslinker (UVP, Upland, CA, USA) with a UV peak at 302 nm. After UVB exposure, the cells were then incubated with a fresh medium containing vehicle or testing compounds at 37 °C for the indicated time. The control group without treatment of the testing compounds was processed in the same way, except for the UVB irradiation.

#### 2.5.5. Measurement of Intracellular Reactive Oxygen Species (ROS) in HaCaT Cells

The intracellular ROS was detected using the cell-permeable 2′,7′-dichlorodihydrofluorescein diacetate (H2DCF-DA; Sigma-Aldrich, St. Louis, MO, USA), which was cleaved by intracellular esterases and converted to the fluorescent product 2′,7′-dichlorofluorescein (DCF) in the presence of ROS. Briefly, the HaCaT cells were pretreated as described above. In the final 30 min of treatment, the cells were loaded with 10 μM H2DCF-DA for 30 min at 37 °C to allow cellular incorporation. The cells were then incubated with PBS for exposure to UVB (40 mJ/cm^2^). The fluorescence with an excitation wavelength of 495 nm and emission of 520 nm was recorded using a fluorescent microplate reader (Bio-Tek Synergy HT, Winooski, VT, USA).

#### 2.5.6. Statistical Analyses

All data are presented as the mean ± standard error of the mean (SEM), derived from at least three independent experiments in triplicate for each treatment group. Statistical significance was analyzed using the Student’s *t*-test (SPSS 13 Inc., Chicago, IL, USA). A value of *p* < 0.05 was considered statistically significant.

### 2.6. ECD Calculations

The lowest energies of the four possibilities of 5*S*,6*S*-monaspurpyridine A (**1**) and 5*R*,6*R*-monaspurpyridine A (**2**) were calculated using Gaussian16 software (Gaussian Inc., Wallingford, CT, USA). The density functional theory (DFT) was applied at the B3LYP/6-311G(d,p) level with IEFPCM in MeOH. The final ECD spectra were generated using GaussSum 3.0 software [69] by applying band shapes with sigma = 0.5 eV. The calculated ECD and experimental ECD curves were illustrated with Excel.

## 3. Results and Discussion

**Figure 1:**
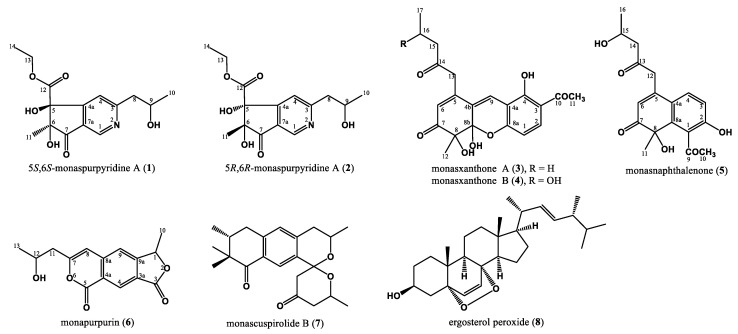
Structures of compounds **1**–**8**.

We focused on the secondary metabolites from the 70% EtOH extract of *M. purpureus* BCRC 38110 fermented on rice in the current study. Through a series of isolation processes, we successfully isolated two new azaphilones, 5*S*,6*S*-monaspurpyridine A (**1**) and 5*R*,6*R*-monaspurpyridine A (**2**), two new xanthonoids, monasxanthones A and B (**3** and **4**), one new naphthalenone, monasnaphthalenone (**5**), one new azaphilone, monapurpurin (**6**), and two known compounds from the 70% EtOH extract of *M. purpureus* BCRC 38110 (Figure 1). The phytochemical spectra of compounds **1**–**6** are available in the Supplementary Materials. In addition, some compounds with sufficient amounts were evaluated for photoprotective activities in the UVB-irradiated HaCaT cells and anti-melanogenic activities in the α-MSH-stimulated B16-F10 cells.

### 3.1. Structure Elucidation of the New Compounds

**Figure 2:**
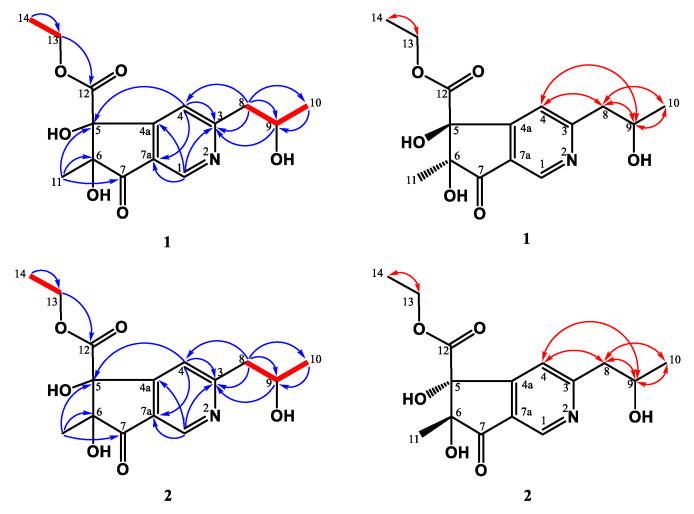
Key ^1^H-^1^H COSY (━), HMBC (H→C), and NOESY (H↔H) correlations of **1** and **2**.

Compounds **1** and **2** were HPLC-separable stereoisomers generated by the cyclopenta[*c*]pyridine with the ethyl acetate group and propanyl-2-ol moieties. They had almost identical spectroscopic properties (HRESIMS, ^1^H, and ^13^C NMR spectra) and physical data (appearance, UV, and IR), except for the opposite optical activity and circular dichroism effect. Both of them were yellowish syrup and established molecular formula C_15_H_19_NO_6_ in the HRESIMS analyses (**1**: 332.11026 [M + Na]^+^; **2**: 332.11020 [M + Na]^+^). The UV spectrum showed maximum absorptions at **1**: 204, 250 (sh), 274 (sh) nm; **2**: 204, 252 (sh), 272 (sh) nm, referring to the typical cyclopentanone and pyridine skeleton [70,71]. Taking the ^1^H NMR spectrum of **1**, for example, three methyl groups [*δ*_H_ 1.07 (t, *J* = 6.0 Hz, H-14)/1.24 (d, *J* = 6.0 Hz, H-10)/1.28 (s, H-11)], two pairs of methylenes [*δ*_H_ 3.00 (m, H-8)/4.07 (m, H-13)], one oxymethine [*δ*_H_ 4.22 (sextet, *J* = 6.0 Hz, H-9)], and two singlet pyridine protons [*δ*_H_ 7.58 (s)/8.87 (s)] were observed. The ^13^C NMR and DEPT spectra indicated the presence of three methyl carbons (*δ*_C_ 14.1, 21.8, and 23.5), one methylene (*δ*_C_ 63.5), two oxygenated quaternary carbons (*δ*_C_ 84.8 and *δ*_C_ 87.1), one ester group (*δ*_C_ 171.8), one *α*,*β*-unsaturated C=O group on the cyclopentanone at *δ*_C_ 203.5, and the pyridine signals (*δ*_C_ 120.8, 129.8, 145.6, 159.5, and 167.6). Based on HMBC correlation (Figure 2) between H-4/C-5, C-7a, and H-1/C-4a, C-7a, the cyclopentanone and pyridine were connected with C-4a and C-7a. The propanyl-2-ol group was confirmed by the COSY correlation (Figure 2) between H-8/H-10 and attached at C-3, based on the HMBC correlation between H-8/C-3, C-4 and H-9/C-3. The methyl group (C-11) occupied C-6 according to the HMBC correlation between H-11/C-5, C-6, C-7. Thus, the remaining ethyl acetate group (C-12/C-13/C-14) was located at quaternary carbon C-5, and two hydroxy groups were connected with C-5 and C-6.

**Figure 3:**
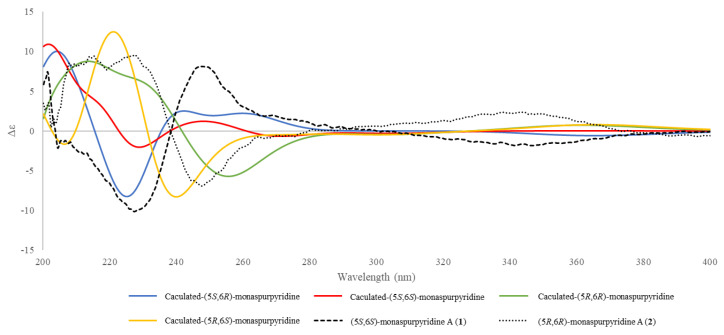
Comparison of the experimental (dash) and calculated (color) ECD spectra of **1** and **2**.

All spectroscopic data of individual compounds **1** and **2** were explained by a diastereomeric character within stereochemical centers C-5/C-6. The ECD spectra of four possibilities were calculated at B3LYP/6-311G(d,p) level with IEFPCM in MeOH [72,73] (Figure 3). After comparison between the experimental spectra of compounds **1** and **2** and the computed electronic circular dichroism (ECD) spectra (Figure 3), compound **1** showed a positive Cotton effect at 200–203 nm and 240–260 nm and a negative Cotton effect at 225–235 nm, similar to those of (5*S*,6*S*)-conformation, and compound **2** showed a positive Cotton effect at 200–220 nm, 220–235, and 310–360 nm and a negative Cotton effect at 240–270 nm, similar to those of (5*R*,6*R*)-conformation. Thus, the absolute configurations of compounds **1** and **2** were assigned and named (5*S*,6*S*)-monaspurpyridine A and (5*R*,6*R*)-monaspurpyridine A, respectively.

**Figure 4:**
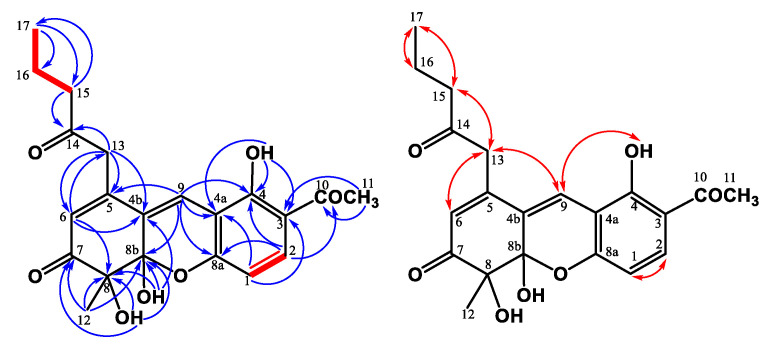
Key ^1^H-^1^H COSY (━), HMBC (H→C), and NOESY (H↔H) correlations of **3**.

Compound **3** was obtained as a yellowish solid, and the molecular formula was assigned as C_21_H_22_O_7_ based on the HRESIMS, indicating 11 indices of hydrogen deficiency (IHD). The ^1^H NMR spectrum of **3** indicated the existence of three methyl groups, including one singlet at *δ*_H_ 1.52 (s, H-12), one triplet at *δ*_H_ 0.94 (t, *J* = 7.2 Hz, H-17), and the acetyl methyl proton at *δ*_H_ 2.59 (s, H-11) (Table 1). It also showed three methylene groups (*δ*_H_ 1.67 (sextet, *J* = 7.2 Hz, H-16), 2.57 (t, *J* = 7.5 Hz, H-15), 3.62 (d, *J* = 16.5 Hz, H-13b), and 3.79 (d, *J* = 16.5 Hz, H-13a)), one *ortho*-coupling aromatic protons (*δ*_H_ 6.65 (d, *J* = 9.0 Hz, H-1)/7.73 (d, *J* = 9.0 Hz, H-2)), two singlet olefinic protons [*δ*_H_ 6.03 (s, H-6)/7.42 (s, H-9)], one chelated hydroxyl [*δ*_H_ 13.3 (s, OH-4)], and two aliphatic alcohols [*δ*_H_ 4.26 (s, OH-8)/4.27 (s, OH-8b)]). In the ^13^C NMR and DEPT spectra, 11 quaternary carbons, 3 primary carbons, 3 secondary carbons, and 4 tertiary carbons could be observed. The low field shift of the carbon signals could also be characterized as three carbonyl groups (*δ*_C_ 198.2 (C-7), 203.2 (C-10), and 205.1 (C-14)) (Table 1). The pentanyl-2-one side chain group was confirmed by the COSY correlation between H-15/H-16/H-17 and the HMBC correlation between H-15/C-14 and H-13/C-14 (Figure 4). The HMBC showed correlations between H-12/C-7, C-8, C-8b, and H-6/C-4b C-8, supporting the existence of 6-mehylcyclohex-2-en-1-one fragment. From the HMBC correlations between OH-8/C-7, C-8, and C-8b and OH-8b/C-4b, C-8, and C-8b, the hydroxy groups were located at C-8 and C-8b. The cross-peak between H-13/C-4b, C-5, and C-6 in the HMBC spectrum could confirm the pentanyl-2-one side chain group was connected with C-5. The HMBC spectrum revealed the correlation between OH-4/C-3, C-4, C-4a, and H-11/C-3, suggesting that the hydroxy group (*δ*_H_ 13.3) was at C-4 and the acetyl group was at C-3. Moreover, the key HMBC correlations of H-9/C-4a, C-5, C-8a, and C-8b verified the junction of the aromatic ring and 6-mehylcyclohex-2-en-1-one at C-9. The above elucidations constructed the chemical skeleton of **1** with 10 IHDs. The last IHD was afforded by the cyclization between C-8a and C-8b through the ether linkage. Therefore, **3** was determined to be a new natural xanthonoid and named monasxanthone A.

**Figure 5:**
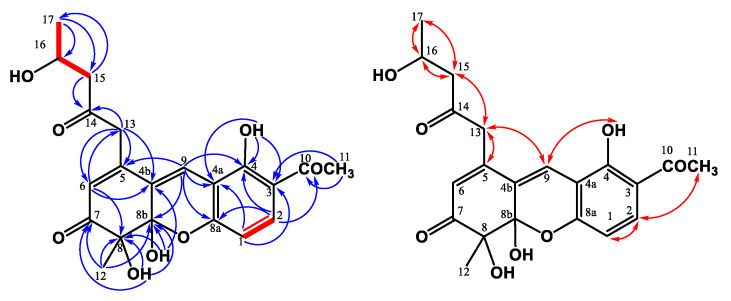
Key ^1^H-^1^H COSY (━), HMBC (H→C), and NOESY (H↔H) correlations of **4**.

Compound **4** was obtained as an optically yellowish solid. The NMR (Table 1), IR, and UV spectra showed that **4** was a xanthonoid analog similar to **3**. Its molecular formula of C_21_H_22_O_8_, one oxygen more than **3**, was determined by HRESIMS. The oxymethine signal at *δ*_H_ 4.29 suggested the existence of a hydroxy group in **4**. The COSY correlation (Figure 5) between H-15 (*δ*_H_ 2.66, 2.75)/H-16 (*δ*_H_ 4.29)/H-17 (*δ*_H_ 1.25), and the HMBC correlation (Figure 5) between H-17/C-16 confirmed that the hydroxy group was located at C-16. This evidence decided that the 4-hydroxypentanyl-2-one moiety was attached to C-5 in **4**, different from the pentanyl-2-one in **3**. On the basis of the above results, the structure of **4** was elucidated and named monasxanthone B.

**Figure 6:**
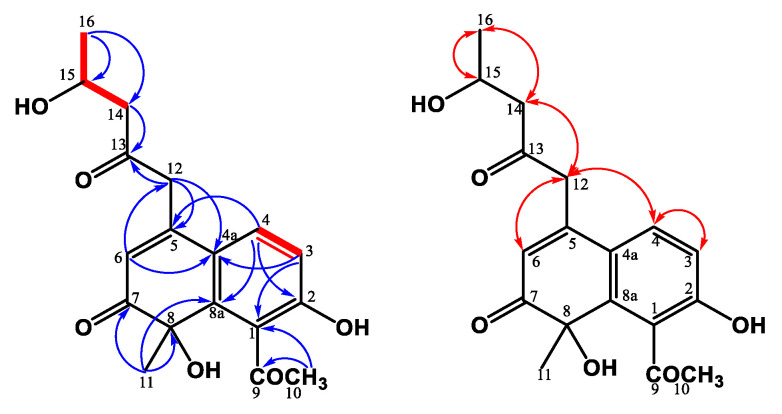
Key ^1^H-^1^H COSY (━), HMBC (H→C), and NOESY (H↔H) correlations of **5**.

Compound **5** was obtained as a yellowish solid. Analysis of the HRESIMS of **5** indicated a molecular formula of C_18_H_20_O_6_, representing nine IHDs. The ^1^H NMR spectrum of **5** was similar to **4**, except for the absence of olefinic proton *δ*_H_ 7.52 in **5**. According to IHDs and the ^1^H NMR spectrum, an aromatic ring in 3 replaced the 2*H*-chromene moiety in **2**. Further analysis of the HMBC correlations (Figure 6) between H-4/C-5, C-8a, H-6/C-4a, and H-11/C-8a showed that the location of the aromatic ring was connected with cyclohexenone ring at C-4a and C-8a. Hence, the 3,6-dihydro-2*H*-pyran moiety in **4** was absent in **5**. The acetyl group was located at C-1 due to the HMBC correlation from H-10 to C-1, and the hydroxy group was connected with C-2 based on the low field shift of C-2 (*δ*_C_ 156.9). Based on the information, the entire structure of **5** was suggested and named monasnaphthalenone.

**Figure 7:**
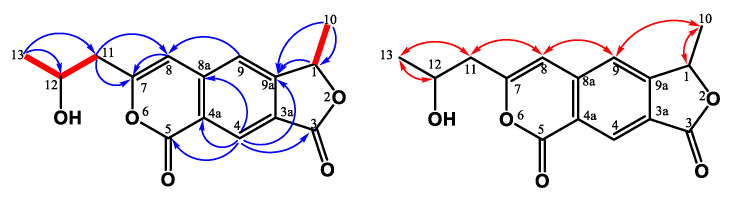
Key ^1^H-^1^H COSY (━), HMBC (H→C), and NOESY (H↔H) correlations of **6**.

Compound **6** was isolated as an optically yellowish solid, and the molecular formula was determined to be C_15_H_14_O_5_ based on the results from HRESIMS. The ^1^H and ^13^C NMR spectra of **6** represented the characteristic skeleton as *Monascus* azaphilone, monascodilone [27], except the propenyl group at C-7 in the monascodilone was hydroxylated to the propanyl-2-ol group in **6**. The COSY correlations (Figure 7) between H-11/H-12/H-13 and the HMBC correlations (Figure 7) between H-13/C-11 and C-12 confirmed the existence of a propanyl-2-ol fragment. Further HMBC correlations between H-11/C-7 and C-8 verified that the propanyl-2-ol fragment was attached to C-7. According to the above data, the structure of monapurpurin (**6**) was confirmed, and it is shown in Figure 7.

By comparing the spectroscopic data ([α]_D_, UV, IR, NMR, and MS) of the known compounds to the previous report, the other known compounds were identified, including one 5′,6′-dihydrospiro[isochromane-1,2′-pyran]-4′(3′*H*)-one derivative, monascuspirolide B (**7**) [55], and one steroid, ergosterol peroxide (**8**) [74].

### 3.2. Photoprotective Activities of Monascuspirolide B (***7***) and Ergosterol Peroxide (***8***) in Human Keratinocytes HaCaT Cells

**Figure 8:**
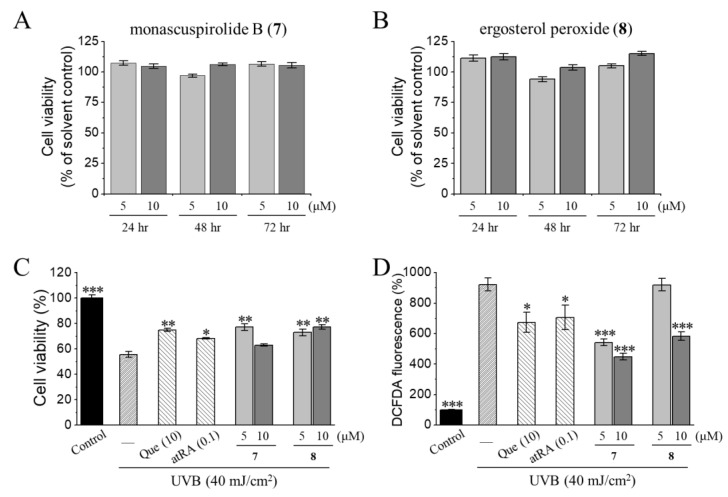
Photoprotective activities of monascuspirolide B (**7**) and ergosterol peroxide (**8**) in HaCaT cells. (**A**,**B**) Monascuspirolide B (**7**) (**A**) and ergosterol peroxide (**8**) (**B**) had no cytotoxicity on the HaCaT cells. After treatment, cells were incubated with 10% volume of alamarBlue^®^ reagent to determine cell viability. (**C**,**D**) Protective effects of monascuspirolide B (**7**) and ergosterol peroxide (**8**) against UVB-induced cell viability loss (**C**) and ROS overproduction (**D**). Cells were pre-incubated with vehicle control or testing compounds for 6 h and then subjected to UVB irradiation. After irradiation, cells were incubated for another 24 h. Intracellular ROS was measured using H2DCF-DA, a cell-permeable fluorescent indicator for intracellular ROS production. Quercetin (Que, 10 μM) and all-*trans* retinoic acid (atRA, 0.1 μM) were used as the reference control. Data were normalized with the basal group and presented as mean ± SEM from at least three independent experiments. * *p* < 0.05. ** *p* < 0.01. *** *p* < 0.005 (compared to the UVB-irradiated group).

We investigated the photoprotective and antioxidant properties of two compounds (**7** and **8**) in sufficient amounts of human keratinocyte HaCaT cells. For assessing the photoprotective activities, quercetin (Que) and all-*trans* retinoic acid (atRA), known for their efficacy in repairing photoaged skin [75], were used as the reference control. Compared with the vehicle control, monascuspirolide B (**7**) and ergosterol peroxide (**8**) at the concentrations of 5 and 10 μM did not affect the cell viability of the HaCaT cells (Figure 8A,B), ensuring the safe concentrations of these two compounds. However, as shown in Figure 8C,D, exposure with 40 mJ/cm^2^ UVB resulted in a significant decrease in cell viability (~50%) and a drastic increase in intracellular ROS levels (~900%) compared with the non-irradiated control group. Pretreatment of monascuspirolide B (**7**) and ergosterol peroxide (**8**) at the concentrations of 5 or 10 μM significantly attenuated the UVB-induced cell viability loss and ROS overproduction, being as effective as quercetin and atRA (Figure 8C,D).

Our results for the cell viability and intracellular ROS levels indicated that the photoprotective effects of monascuspirolide B (**7**) and ergosterol peroxide (**8**) at non-cytotoxic concentrations involved, at least partially, the attenuation of oxidative stress, which is a major contributing factor of UVB-induced photodamage. It was shown that ergosterol peroxide (**8**) exhibited antioxidant activity on the inhibition of lipid peroxidation of rat liver microsomes [76] and a photoprotective effect against the UVA-induced gene expression of keratinocytes [77]. The present study is the first to reveal the photoprotective and antioxidant properties of monascuspirolide B (**7**) and ergosterol peroxide (**8**) against UVB-induced photodamage of human keratinocytes. However, whether the photoprotective mechanisms of monascuspirolide B (**7**) and ergosterol peroxide (**8**) involve the direct scavenging of free radicals or the modulation of antioxidant defense mechanisms requires further investigation. Future directions will focus on the potentials of these two compounds for alleviating oxidative stress-related inflammatory damage to the skin.

### 3.3. Anti-Melanogenic Activities of Ergosterol Peroxide (**8**) in Mouse Melanoma B16-F10 Cells

**Figure 9:**
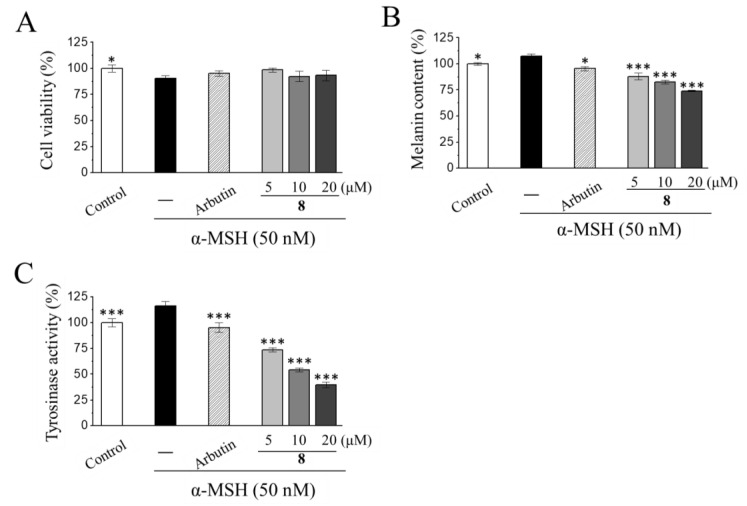
Anti-melanogenic activities of ergosterol peroxide (**8**) in murine melanoma B16-F10 cells. Cells were grown with (induction) or without (basal) 50 nM α-melanocyte-stimulating hormone (α-MSH) for 24 h to induce melanogenesis and then incubated with vehicle control or 5, 10, or 20 μM of the testing compound for another 48 h. After treatment, cells were incubated with 10% volume of alamarBlue^®^ reagent to determine cell viability (**A**), then harvested by trypsinization for analyzing the (**B**) melanin content and (**C**) tyrosinase activity. Arbutin (1 μM) was used as the reference control. Data were normalized with the basal group and presented as mean ± SEM from at least three independent experiments. * *p* < 0.05. *** *p* < 0.005, compared to the α-MSH-induction control group.

The anti-melanogenic potential of two compounds (**7** and **8**) in sufficient amounts was further evaluated. In the melanin content and tyrosinase activity assays, the B16-F10 cells were stimulated with 50 nM α-MSH for 24 h and then treated with either a vehicle or testing compounds. Kojic acid and arbutin, which are potent tyrosinase inhibitors [57,60,66], were used as reference controls. Both the monascuspirolide B (**7**) and ergosterol peroxide (**8**) at a concentration of 20 μM did not significantly affect cell viability, as compared with the α-MSH-induced B16-F10 cells (Table 2). Importantly, ergosterol peroxide (**8**) at a concentration ranging from 5 to 20 μM exhibited a concentration-dependent inhibition of α-MSH-induced melanin production and tyrosinase activity without significant effects on cell viability (Figure 9A–C). In contrast, monascuspirolide B (**7**) at 20 μM did not affect the melanin production or tyrosinase activity in the α-MSH-treated B16-F10 cells (Table 2).

The combined results for the cell viability, cellular melanin contents, and cellular tyrosinase activity in the B16-F10 cells indicated that monascuspirolide B (**7**) and ergosterol peroxide (**8**) decreased the melanin contents and tyrosinase activities of the melanocytes without affecting cell viability. Therefore, the anti-melanogenic effects of monascuspirolide B (**7**) and ergosterol peroxide (**8**) at non-cytotoxic concentrations were, at least partially, contributed by the inhibition of tyrosinase activities. To our knowledge, this is the first report on the anti-melanogenic effects of monascuspirolide B (**7**). Additionally, our results on ergosterol peroxide (**8**) were consistent with previous investigations in the model of B16 10F7 mouse melanoma cells, showing the inhibitory effects of ergosterol peroxide (**8**) on the cellular melanin content and expressions of tyrosinase-related enzyme TRP-1 [78]. However, whether the anti-melanogenic mechanisms of monascuspirolide B (**7**) and ergosterol peroxide (**8**) involves direct inhibition of the enzymatic activities or expressions of tyrosinase [79] or the downregulation of melanogenesis transcription factors and signaling pathways [80] requires further investigation.

## 4. Conclusions

In this study, six new compounds along with two known compounds were isolated from *M. purpureus* BCRC 38110. Among them, 5*S*,6*S*-monaspurpyridine A (**1**) and (5*R*,6*R*)-monaspurpyridine A (**2**) are unpresented 5*H*-cyclopenta[*c*]pyridine type azaphilones bearing the N atom. Only one research work has discussed the sensitivity of a related skeleton compound for specific subtypes of nicotinic acetylcholine receptors [81]. The skeleton of new C_6_-C_1_-C_6_ type xanthonoids (monasxanthones A and B (**3** and **4**)) and naphthalen-2-one (monasnaphthalenone (**5**)) were rarely reported in *Monascus* sp. [82,83,84]. Xanthonoids are yellow pigments restricted only to a few families of higher plants, some fungi, and lichens. Recently, xanthonoids have gained attention for their diverse bioactivities, such as cardiovascular protective, antiprotozoal, antioxidant, and anti-tumor effects [84]. The functional group diversity and pharmacological variety of xanthonoids flagged the importance of the investigation of xanthonoids [85]. Our findings from the current study can provide another natural source of xanthonoid derivatives for further applications. Compared with naphthalen-1-one type compounds, the naphthalen-2-one type compound (**5**) is rarely seen. Previous studies have identified a broad spectrum of biological activities of naphthalenes, including anti-inflammatory, antiprotozoal, cytotoxic, anti-oxidant, anti-microbial, and anti-platelet effects, supporting the potential for further investigation and applictaions of naphthalenes [86]. Usually, azaphilone pigment (monascin and ankaflavin) is a major skeleton in *Monascus* sp. To the best of our knowledge, this study demonstrates the first findings on xanthonoids (**3** and **4**) and naphthalenone (**5**) from *Monascus* sp. Monapurpurin (**6**) is a linear azaphilone bearing furanone and pyranone, and it is rarely seen in naturally occurring compounds. The most similar compound to **6** is monascodilone, which was also isolated from *M. purpureus*. However, its bioactivity was not examined [27]. The phytochemical results in the present study not only shed light on the structural diversity of *M. purpureus* but also uncovered different types of compounds from the natural source. Moreover, our results from two different models of skin disorders also demonstrated the dermoprotective potential of *M. purpureus* BCRC 38110 for the development of cosmeceutical products. In particular, monascuspirolide B (**7**) possesses photoprotective activity, and ergosterol peroxide (**8**) exhibits photoprotective and anti-melanogenic activities. This study provides substantive evidence for developing dietary supplements or functional foods of red yeast rice for skin photoaging and paves the way for repurposing the ancient wisdom of *Monascus*-fermented products.

## Figures and Tables

**Table 1:** ^1^H (600 MHz) and ^13^C (150 MHz) NMR data of monasxanthones A and B (**3** and **4**) in CDCl_3_.

Position	Monasxanthone A (3)		Monasxanthone B (4)	
	*δ*_H_ (m, *J* in Hz)	*δ*_C_	*δ*_H_ (m, *J* in Hz)	*δ*_C_
1	6.65 (dd, 9.0, 0.6)	108.7	6.66 (dd, 9.0, 0.6)	108.8
2	7.73 (d, 9.0)	133.9	7.73 (d, 9.0)	134.0
3		114.6		114.6
4		161.3		161.2
4a		109.4		109.4
4b		125.5		125.3
5		149.5		149.2
6	6.03 (s)	123.1	6.03 (d, 0.5)	123.3
7		198.2		198.2
8		79.1		79.1
8a		157.2		157.2
8b		97.0		97.0
9	7.42 (s)	123.5	7.52 (s)	123.8
10		203.2		203.2
11	2.59 (s)	26.3	2.59 (s)	26.3
12	1.52 (s)	23.0	1.53 (s)	23.0
13	3.62 (d, 16.5)	46.9	3.67 (d, 17.1)	48.0
	3.79 (d, 16.5)		3.90 (d, 17.1)	
14		205.1		205.9
15	2.57 (t, 7.5)	44.6	2.66 (dd, 16.4, 3.3)	50.5
			2.75 (dd, 16.4, 9.0)	
16	1.67 (sextet, 7.2)	17.2	4.29 (m)	64.6
17	0.94 (t, 7.2)	13.6	1.25 (d, 6.6)	22.97
OCH_3_-16				
OH-4	13.3 (s)		13.4 (s)	
OH-8	4.26 (s)		4.25 (s)	
OH-8b	4.27 (s)		4.28 (s)	
OH-16			2.60 (br s)	

**Table 2:** Anti-melanogenic activities of test compounds in the mouse melanoma cell line B16-F10.

Treatments	Cell Viability *^a^* (% of Basal Group)	Melanin Content *^a^* (% of Basal Group)	Tyrosinase Activity *^a^* (% of Basal Group)
α-MSH induction	99.3 ± 3.6	106.8 ± 3.7	135.5 ± 1.8
Monascuspirolide B (**7**)	106.9 ± 2.9	96.2 ± 6.0	137.0 ± 1.5
Ergosterol peroxide (**8**)	98.6 ± 0.9	55.0 ± 0.7 *	73.7 ± 1.9 *
Kojic acid *^b^*	97.4 ± 0.7	88.0 ± 2.0 *	109.7 ± 1.9 *

*^a^* Mouse melanoma cell line B16-F10 cells were grown with (induction) or without (basal) 50 nM α-melanocyte-stimulating hormone (α-MSH) for 24 h to induce melanogenesis and then incubated with 20 μM of test compounds for another 48 h. After treatment, cells were incubated with 10% volume of alamarBlue^®^ reagent to determine cell viability, then harvested by trypsinization for analyzing tyrosinase activity and melanin content. *^b^* Kojic acid (1 mM) was used as a reference control. Data were normalized with the basal group and presented as mean ± SEM from at least three replicate wells. * *p* < 0.05, compared to the induction control group.

## Supplementary Materials

The following are available online at https://www.mdpi.com/article/10.3390/jof7080619/s1, including the phytochemical spectra of compounds **1**–**6**.
