# Supplementary material for: Investigations into Chemical Components from Monascus purpureus with Photoprotective and Anti-Melanogenic Activities
Source: J Fungi (Basel). 2021 Jul 29;7(8):619. doi: 10.3390/jof7080619 (PMC8396976; doi:10.3390/jof7080619)
Supplement: Supplementary file 1 [file jof-07-00619-s001.zip › jof-1270802 suppl r1.pdf]

Investigations into chemical components from *Monascus purpureus* with photoprotective and anti-melanogenic activities

Ho-Cheng Wu[1,2], Yih-Fung Chen[1,2,3], Ming-Jen Cheng[4], Ming-Der Wu[4], Yen-Lin Chen[4] and Hsun-Shuo Chang[1,2,3,*]

[1] Graduate Institute of Natural Products, College of Pharmacy, Kaohsiung Medical University, Kaohsiung 807, Taiwan; duncanwu762001@gmail.com (H.-C.W.); yihfungchen@kmu.edu.tw (Y.-F.C.)

[2] School of Pharmacy, College of Pharmacy, Kaohsiung Medical University, Kaohsiung 807, Taiwan

[3] Department of Medical Research, Kaohsiung Medical University Hospital, Kaohsiung 807, Taiwan

[4] Bioresource Collection and Research Center (BCRC), Food Industry Research and Development Institute (FIRDI), Hsinchu 300, Taiwan; cmj@firdi.org.tw (M.-J.C.); wmd@firdi.org.tw (M.-D.W.); alc@firdi.org.tw (Y.-L.C.)

* Correspondence: hschang@kmu.edu.tw; Tel: +886-7312-1101 ext. 2664; Fax: +886-7321-0683 (H.-S.C.)

Appendix A. Supplementary data

Figure S1. ^{1}H NMR spectrum of 5*S*,6*S*-monaspurpyridine A (**1**) in CD_3OD at 600 MHz 4
Figure S2. ^{13}C NMR spectrum of 5*S*,6*S*-monaspurpyridine A (**1**) in CD_3OD at 125 MHz 4
Figure S3. DEPT spectrum of 5*S*,6*S*-monaspurpyridine A (**1**)........ 5
Figure S4. COSY spectrum of 5*S*,6*S*-monaspurpyridine A (**1**) 5
Figure S5. HMBC spectrum of 5*S*,6*S*-monapurpyridine A (**1**) 6
Figure S6. NOESY spectrum of 5*S*,6*S*-monaspurpyridine A (**1**) 6
Figure S7. HRESIMS spectrum of 5*S*,6*S*-monapurpyridine A (**1**)........ 7
Figure S8. CD spectrum of 5*S*,6*S*-monaspurpyridine A (**1**) 7
Figure S9. ^{1}H NMR spectrum of 5*R*,6*R*-monaspurpyridine A (**2**) in CD_3OD at 600 MHz 8
Figure S10. ^{13}C NMR spectrum of 5*R*,6*R*-monaspurpyridine A (**2**) in CD_3OD at 125 MHz 8
Figure S11. DEPT spectrum of 5*R*,6*R*-monaspurpyridine A (**2**)........ 9
Figure S12. COSY spectrum of 5*R*,6*R*-monaspurpyridine A (**2**) 9
Figure S13. HMBC spectrum of 5*R*,6*R*-monaspurpyridine A (**2**)........ 10
Figure S14. NOESY spectrum of 5*R*,6*R*- monaspurpyridine A (**2**) 10
Figure S15. HRESIMS spectrum of 5*R*,6*R*-monaspurpyridine A (**2**) 11
Figure S16. CD spectrum of 5*R*,6*R*-monaspurpyridine A (**2**) 11
Figure S17. ^{1}H NMR spectrum of monasxanthone A (**3**) in $CDCl_3$ at 600 MHz 12
Figure S18. ^{13}C NMR spectrum of monasxanthone A (**3**) in $CDCl_3$ at 125 MHz........ 12
Figure S19. DEPT spectrum of monasxanthone A (**3**) 13
Figure S20. COSY spectrum of monasxanthone A (**3**)........ 13
Figure S21. HMBC spectrum of monasxanthone A (**3**) 14
Figure S22. NOESY spectrum of monasxanthone A (**3**) 14
Figure S23. HRESIMS spectrum of monasxanthone A (**3**)........ 15
Figure S24. ^{1}H NMR spectrum of monasxanthone B (**4**) in $CDCl_3$ at 600 MHz 15
Figure S25. ^{13}C NMR spectrum of monasxanthone B (**4**) in $CDCl_3$ at 125 MHz 16
Figure S26. DEPT spectrum of monasxanthone B (**4**)........ 16
Figure S27. COSY spectrum of monasxanthone B (**4**)........ 17
Figure S28. HMBC spectrum of monasxanthone B (**4**) 17
Figure S29. NOESY spectrum of monasxanthone B (**4**)........ 18
Figure S30. HRESIMS spectrum of monasxanthone B (**4**)........ 18
Figure S31. ^{1}H NMR spectrum of monasnaphthalenone (**5**) in acetone-d_6 at 600 MHz 19

Figure S32. ^{13}C NMR spectrum of monasnaphthalenone (**5**) in acetone-d_6 at 125 MHz 19
Figure S33. COSY spectrum of monasnaphthalenone (**5**) 20
Figure S34. HMBC spectrum of monasnaphthalenone (**5**) 20
Figure S35. NOESY spectrum of monasnaphthalenone (**5**) 21
Figure S36. HRESIMS spectrum of monasnaphthalenone (**5**) 21
Figure S37. ^{1}H NMR spectrum of monapurpurin (**6**) in acetone-d_6 at 600 MHz 22
Figure S38. ^{13}C NMR spectrum of monapurpurin (**6**) in acetone-d_6 at 125 MHz 22
Figure S39. COSY spectrum of monapurpurin (**6**) 23
Figure S40. HMBC spectrum of monapurpurin (**6**) 23
Figure S41. NOESY spectrum of monapurpurin (**6**) 24
Figure S42. HRESIMS spectrum of monapurpurin (**6**) 24

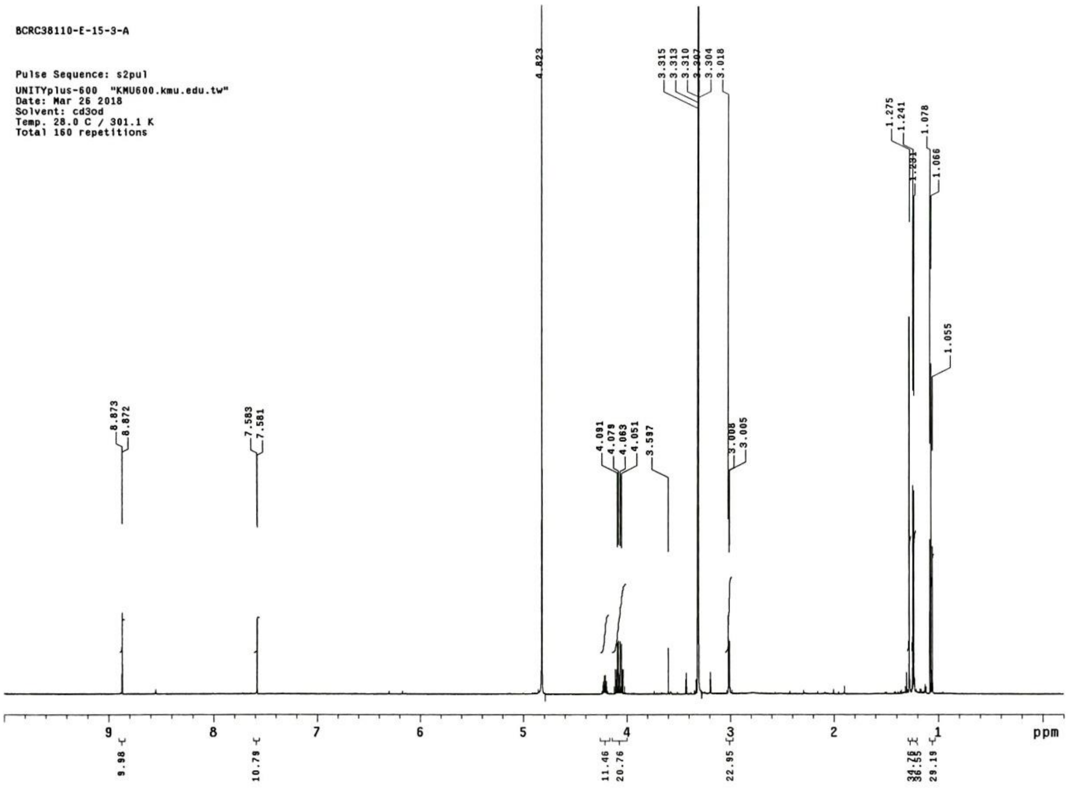

Figure S1. ^1H NMR spectrum of 5*S*,6*S*-monaspurpyridine A (**1**) in CD_3OD at 600 MHz

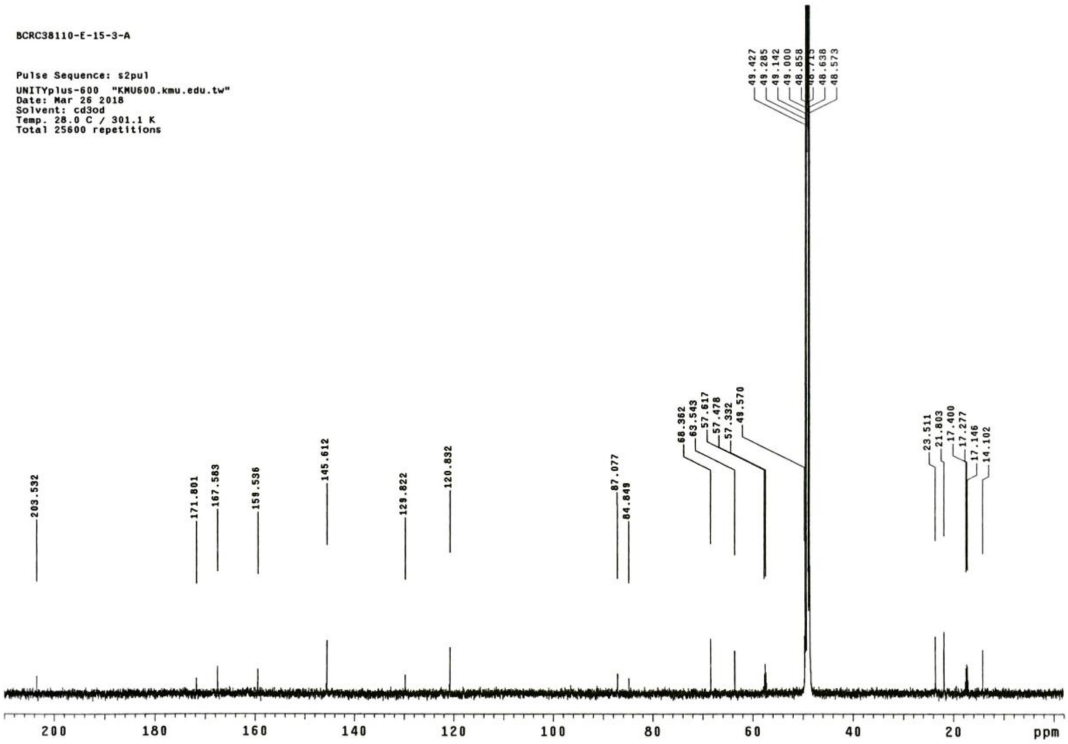

Figure S2. ^{13}C NMR spectrum of 5*S*,6*S*-monaspurpyridine A (**1**) in CD_3OD at 125 MHz

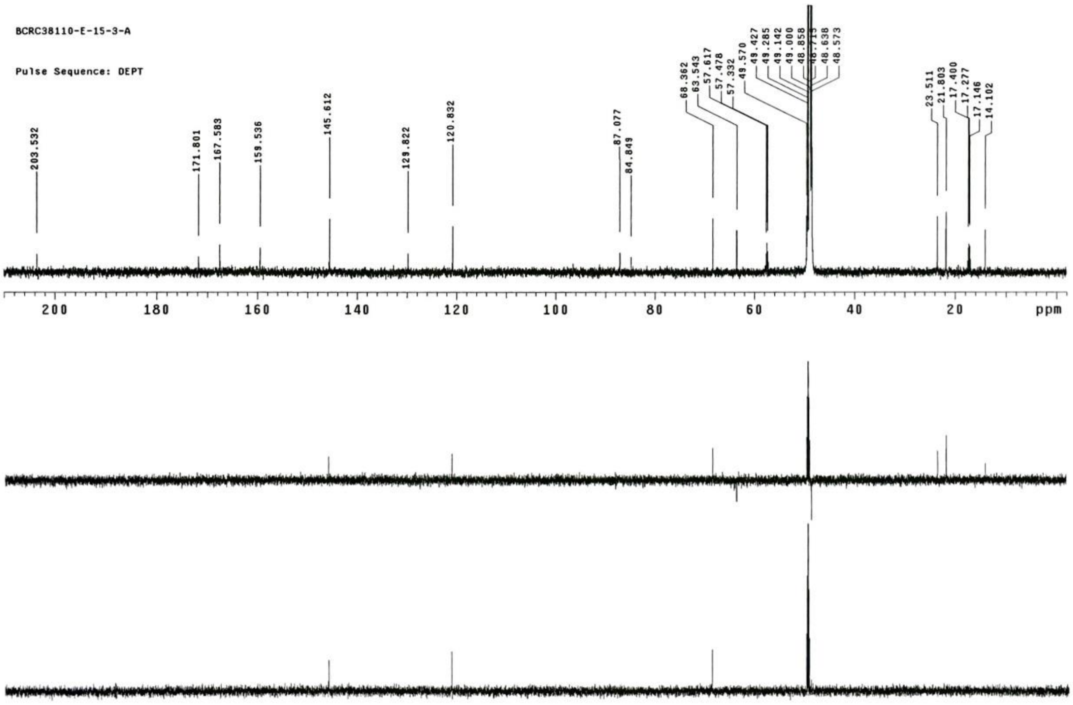

Figure S3. DEPT spectrum of 5*S*,6*S*-monaspurpyridine A (**1**)

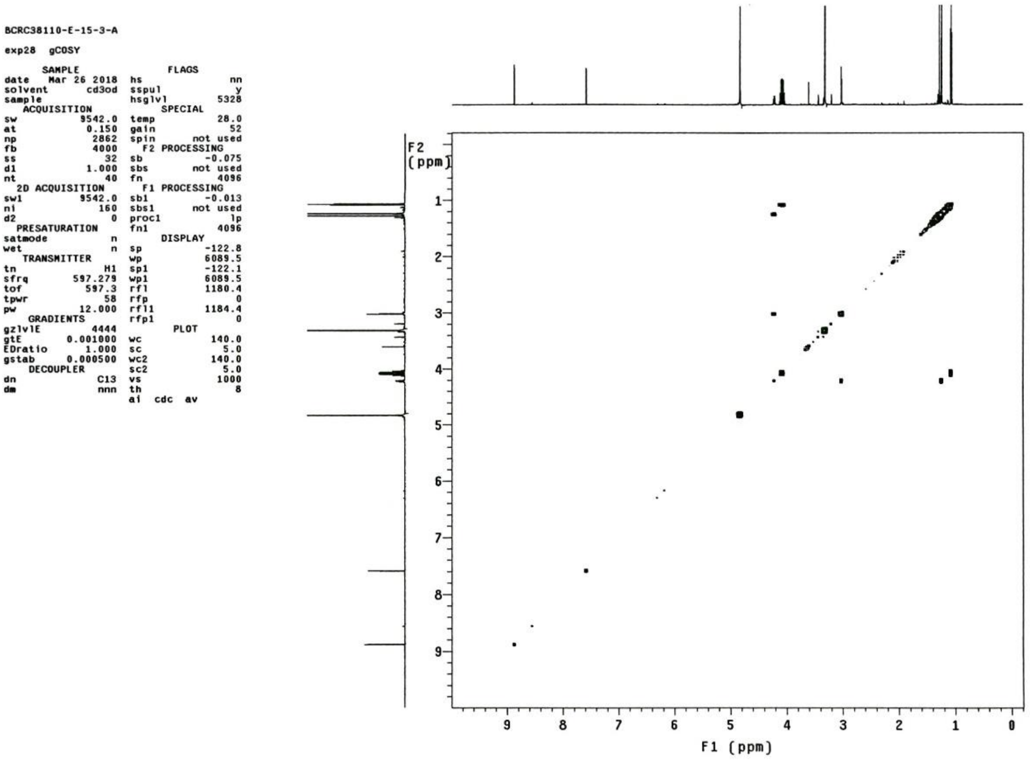

Figure S4. COSY spectrum of 5*S*,6*S*-monaspurpyridine A (**1**)

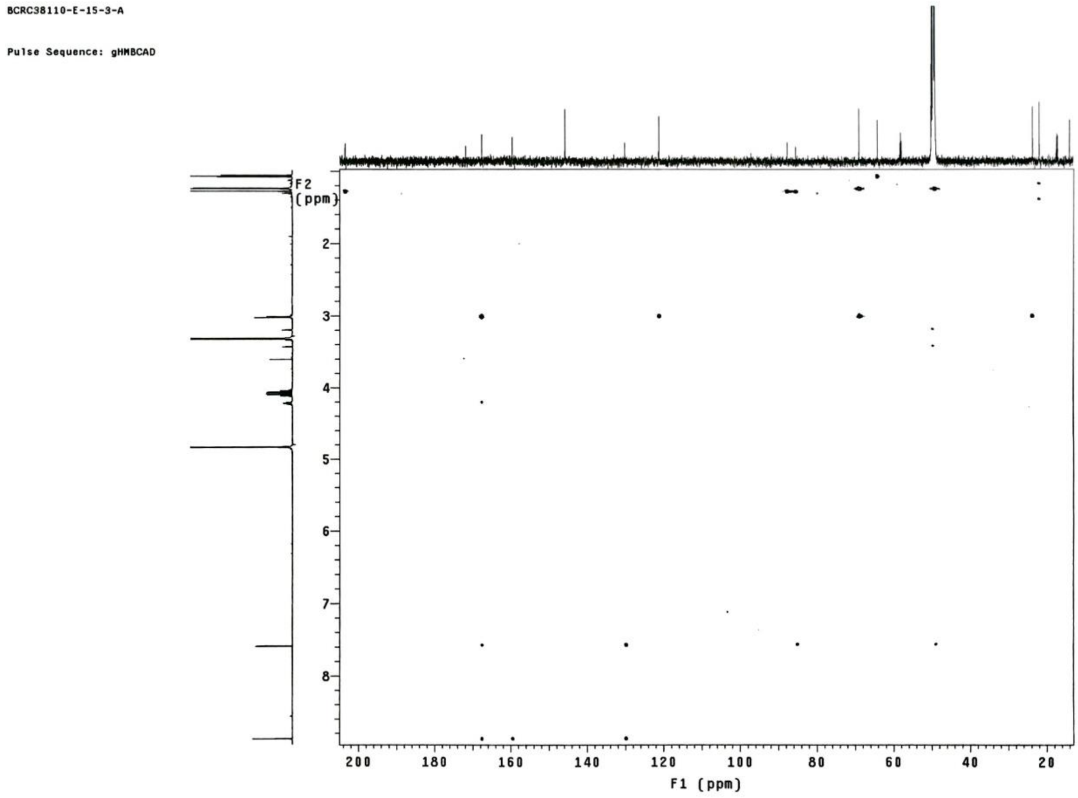

Figure S5. HMBC spectrum of 5*S*,6*S*-monapurpyridine A (**1**)

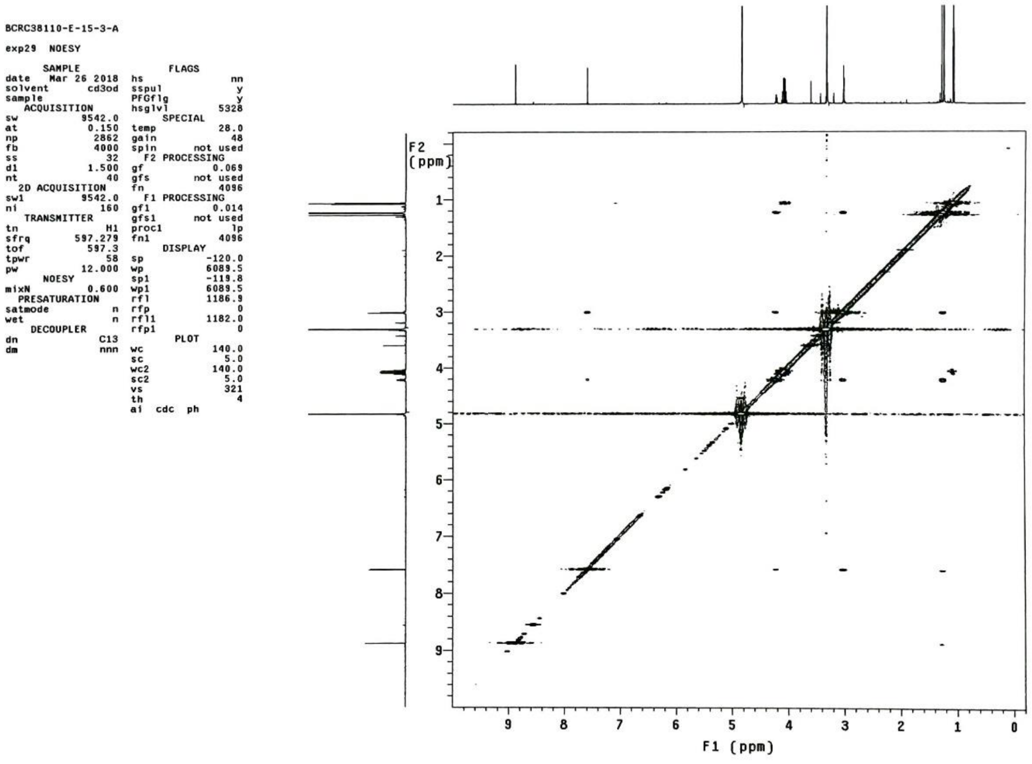

Figure S6. NOESY spectrum of 5*S*,6*S*-monaspurpyridine A (**1**)

Mass Spectrum SmartFormula Report

Analysis Info

Analysis Name	D:\Data\f7\BCRC38110E153A_000005.d	5/11/2018 3:36:13 PM
Method	broadband first signal	Operator: YU HSIAO-CHING
Sample Name	BCRC38110-E-15-3-A	Instrument: BRUKER FT-MS solariX
Comment	ESI Positive	

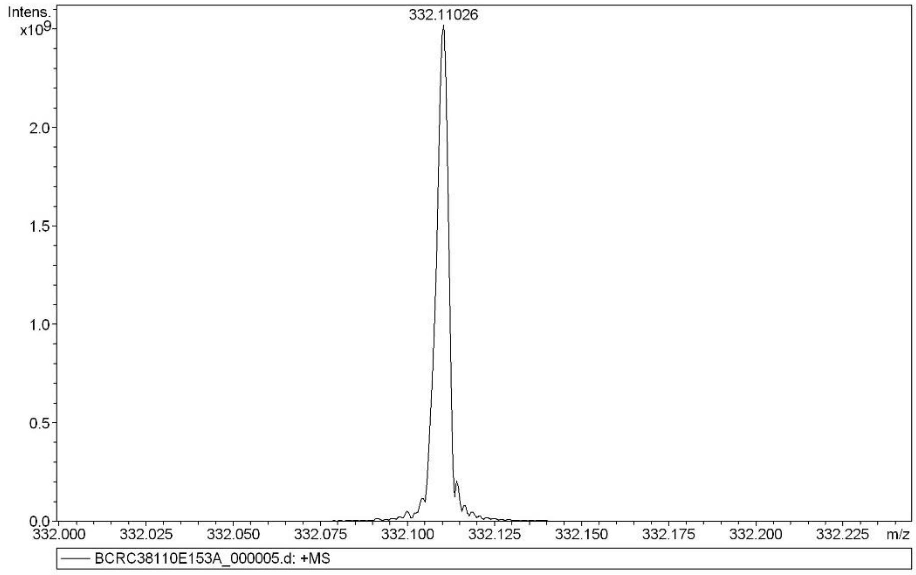

Meas. m/z	#	Formula	Score	m/z	err [mDa]	err [ppm]	mSigma	rdb	e⁻ Conf	N-Rule
332.11026	1	C 15 H 19 N Na O 6	100.00	332.11046	0.20	0.59	10.1	6.5	even	ok

Figure S7. HRESIMS spectrum of 5*S*,6*S*-monapurpyridine A (**1**)

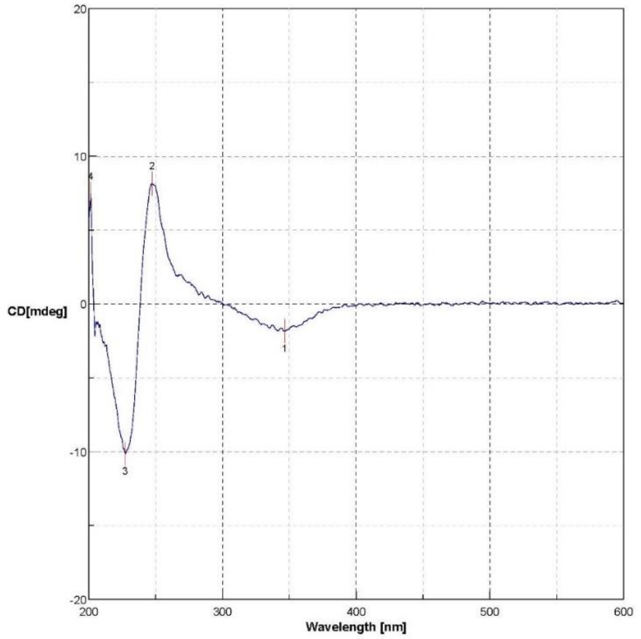

Date/Time	2018/7/4 9:53下午
Operator	User
File Name	BCRC38110-E-15-3-A(smooth).jws
Sample Name	
Comment	

No.	nm	CD[mdeg]	No.	nm	CD[mdeg]	No.	nm	CD[mdeg]
1	346.6	-1.84031	2	247.2	8.1544	3	227.4	-10.1339
4	201.4	7.45978						

Figure S8. CD spectrum of 5*S*,6*S*-monaspurpyridine A (**1**)

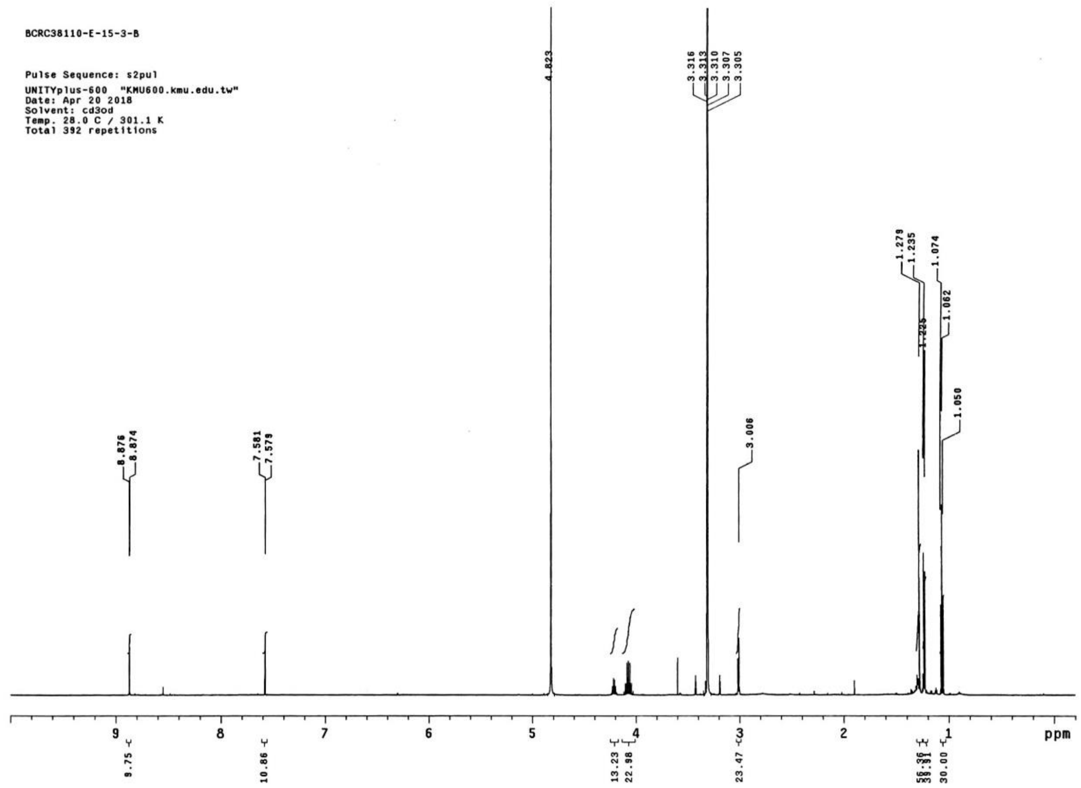

Figure S9. ^{1}H NMR spectrum of 5*R*,6*R*-monaspurpyridine A (**2**) in CD_3OD at 600 MHz

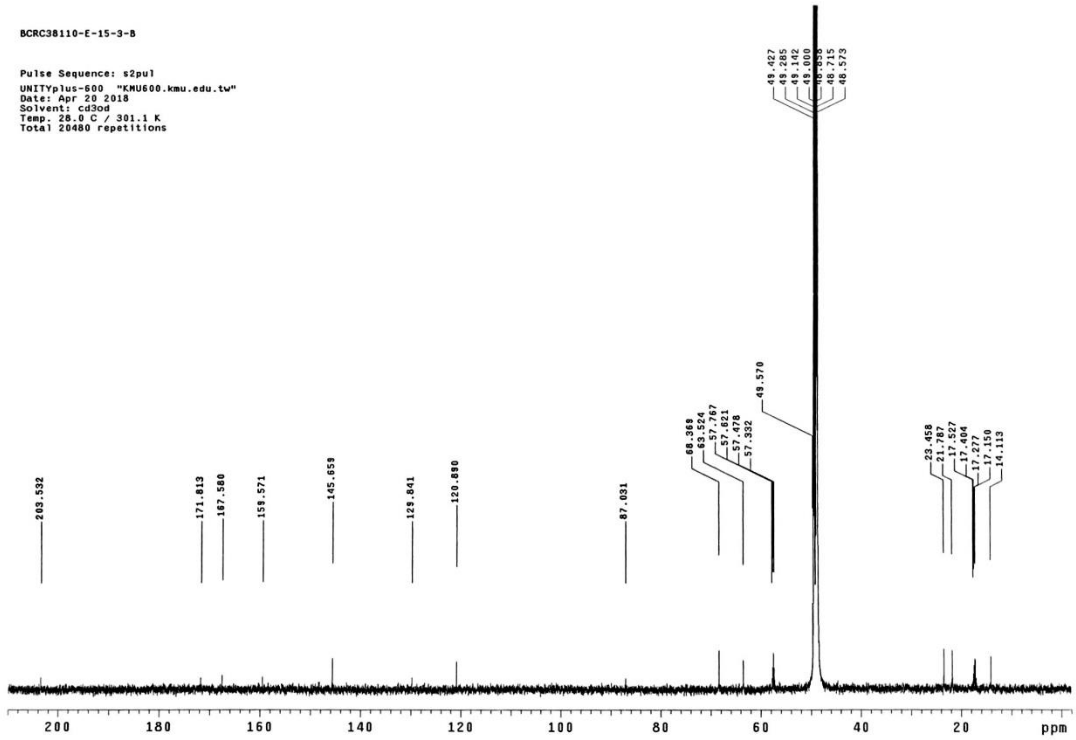

Figure S10. ^{13}C NMR spectrum of 5*R*,6*R*-monaspurpyridine A (**2**) in CD_3OD at 125 MHz

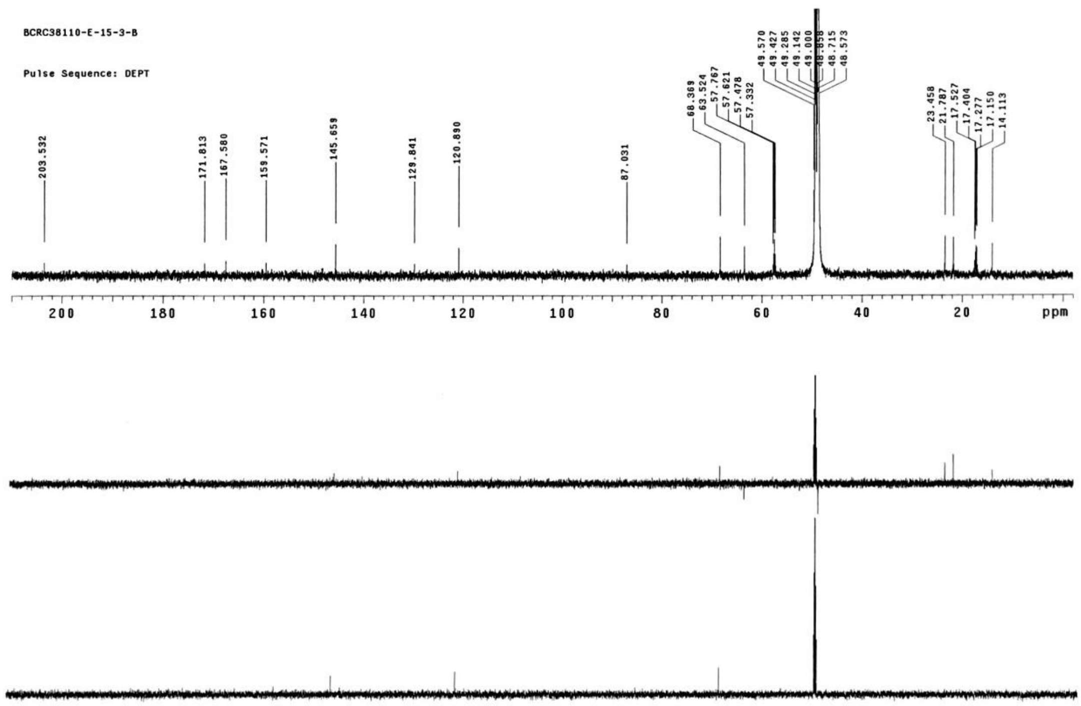

Figure S11. DEPT spectrum of 5*R*,6*R*-monaspurpyridine A (**2**)

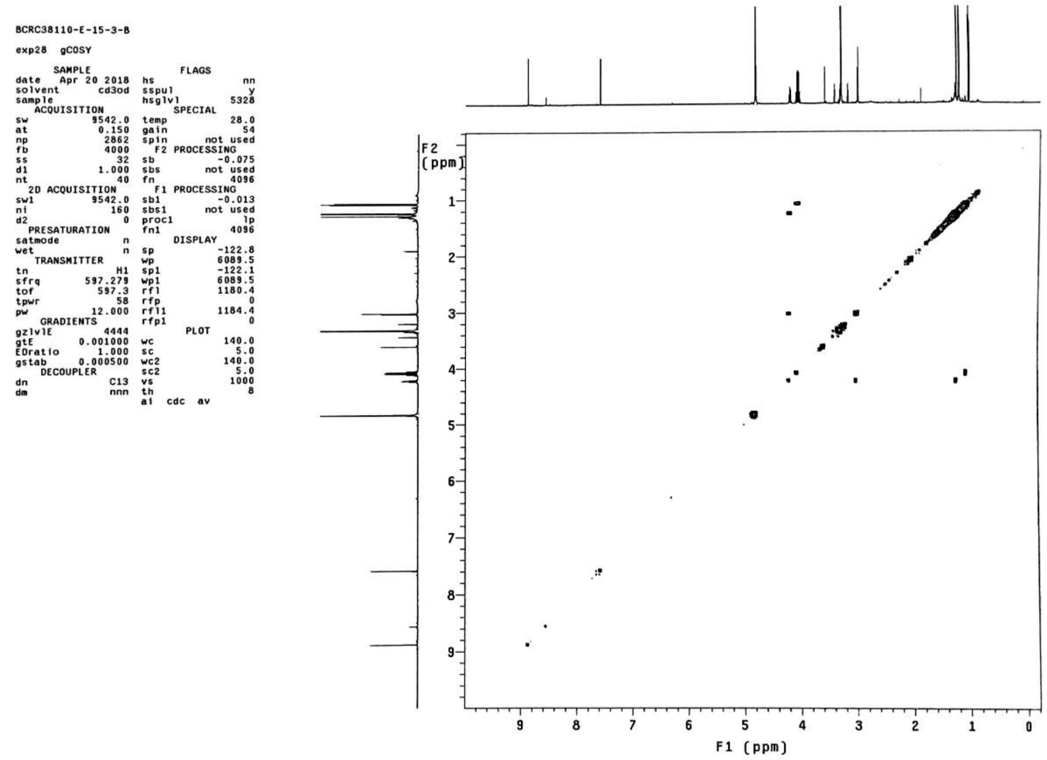

Figure S12. COSY spectrum of 5*R*,6*R*-monaspurpyridine A (**2**)

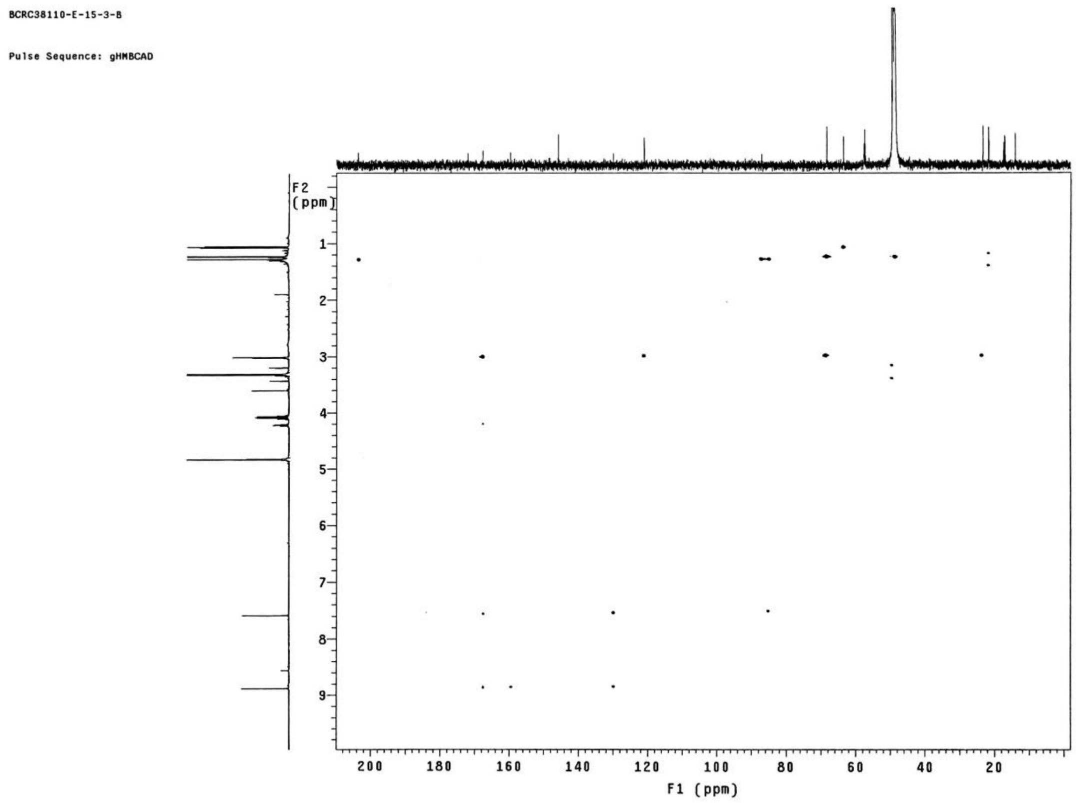

Figure S13. HMBC spectrum of 5*R*,6*R*-monaspurpyridine A (**2**)

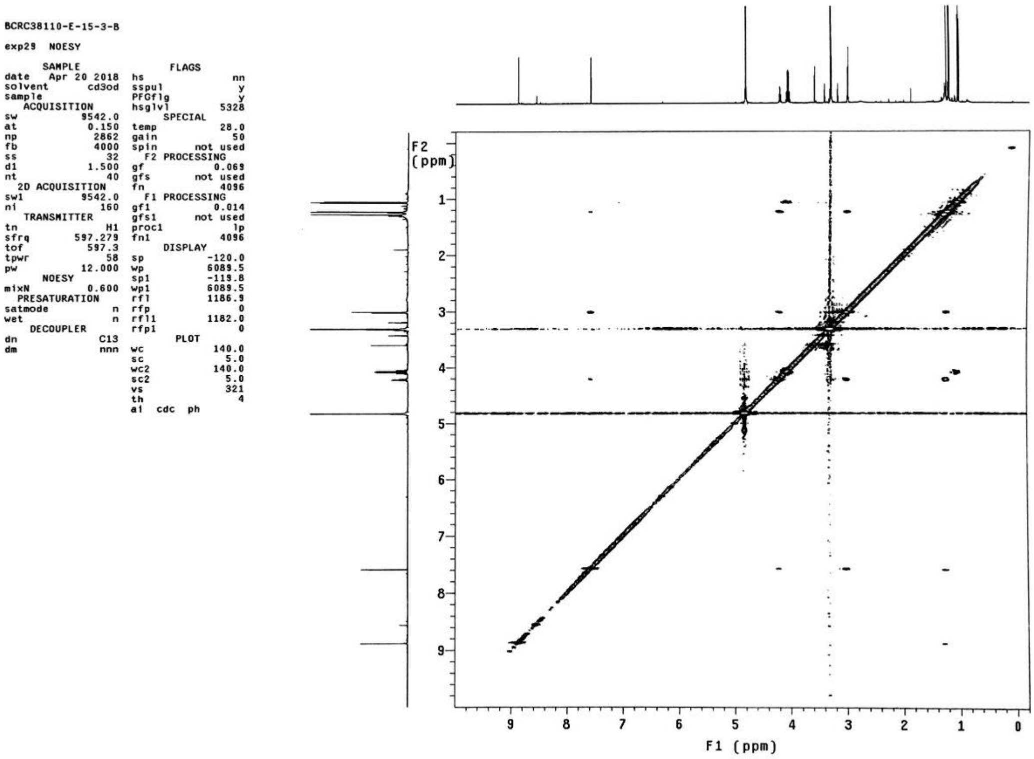

Figure S14. NOESY spectrum of 5*R*,6*R*- monaspurpyridine A (**2**)

Mass Spectrum SmartFormula Report

Analysis Info

Analysis Name	D:\Data\f7\BCRC38110E153B_000005.d	5/11/2018 4:08:05 PM
Method	broadband first signal	Operator: YU HSIAO-CHING
Sample Name	BCRC38110-E-15-3-B	Instrument: BRUKER FT-MS solariX
Comment	ESI Positive	

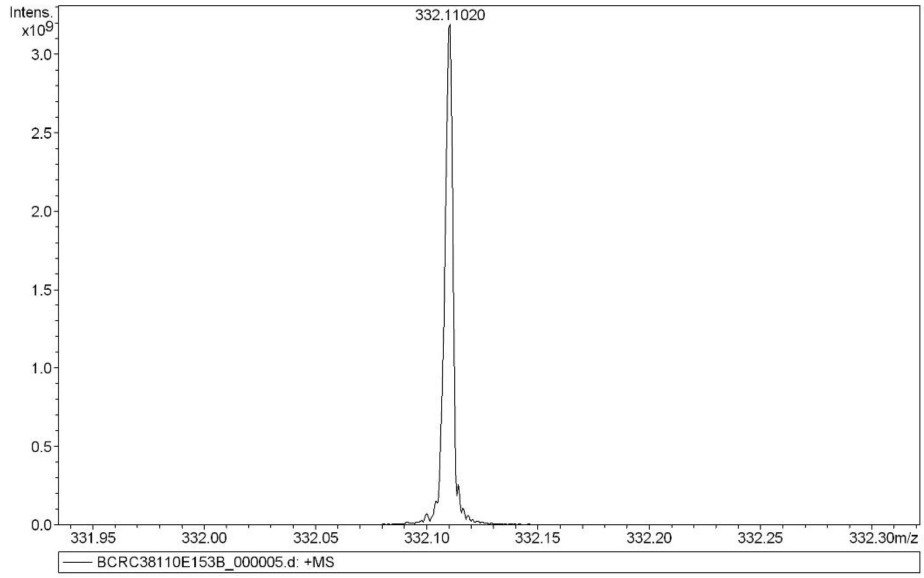

Meas. m/z	#	Formula	Score	m/z	err [mDa]	err [ppm]	mSigma	rdb	e⁻ Conf	N-Rule
332.11020	1	C 15 H 19 N Na O 6	100.00	332.11046	0.26	0.78	14.0	6.5	even	ok

Figure S15. HRESIMS spectrum of 5*R*,6*R*-monaspurpyridine A (**2**)

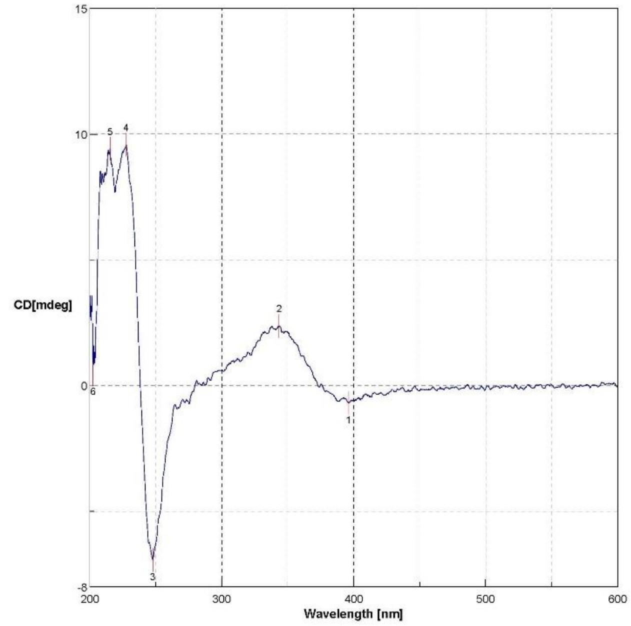

Date/Time	2018/7/4 10:01 下午
Operator	User
File Name	BCRC38110-E-15-3-B(smooth).jws
Sample Name	
Comment	

No.	nm	CD[mdeg]	No.	nm	CD[mdeg]	No.	nm	CD[mdeg]
1	396.1	-0.683127	2	343.4	2.37172	3	247.7	-6.92545
4	227.6	9.57412	5	215.4	9.41946	6	202.5	0.443001

Figure S16. CD spectrum of 5*R*,6*R*-monaspurpyridine A (**2**)

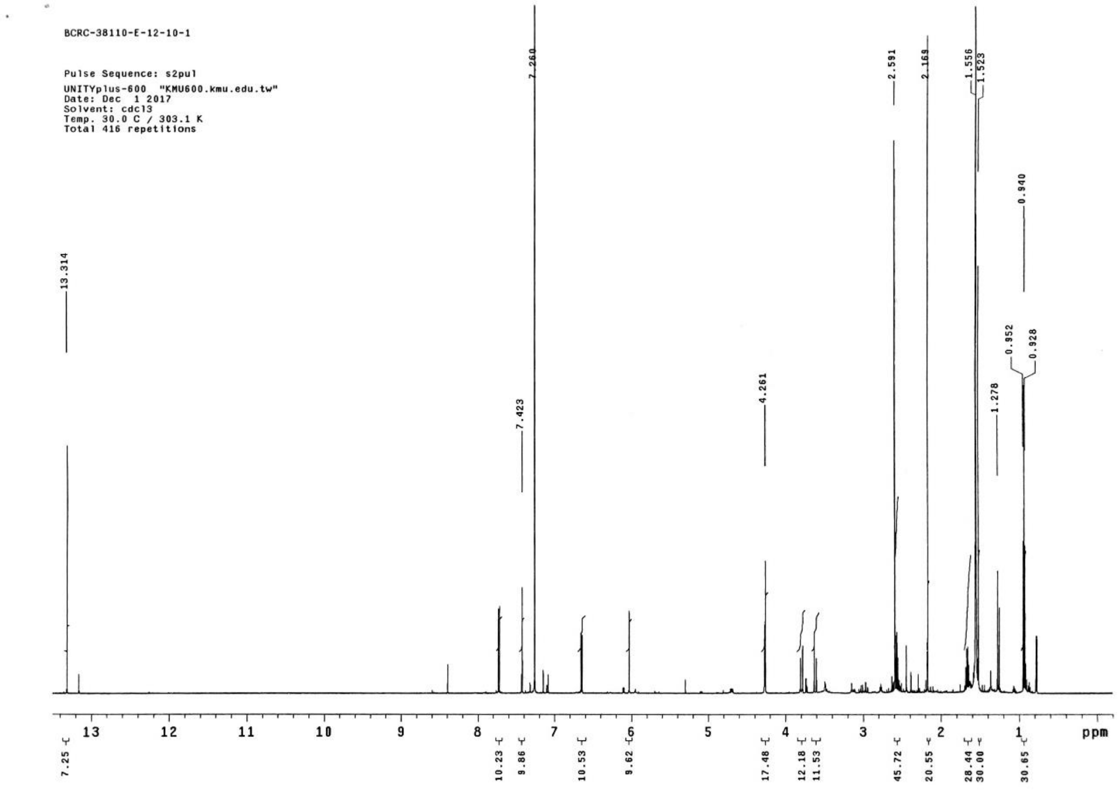

Figure S17. ^{1}H NMR spectrum of monasxanthone A (**3**) in $CDCl_3$ at 600 MHz

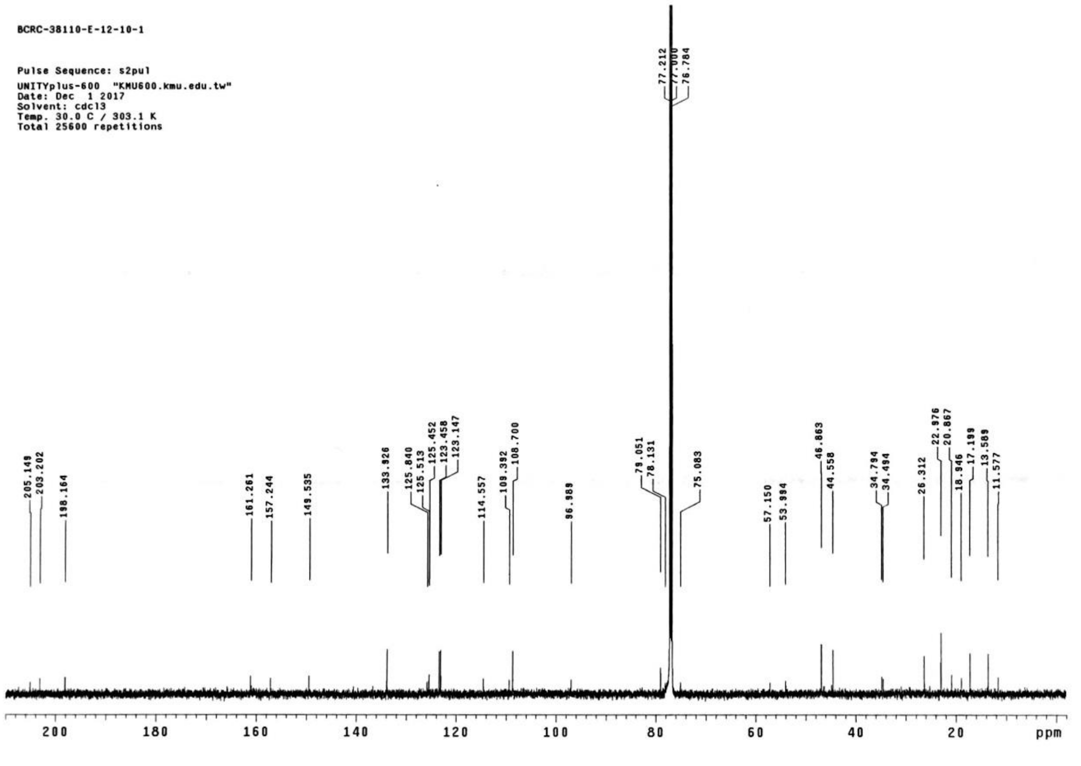

Figure S18. ^{13}C NMR spectrum of monasxanthone A (**3**) in $CDCl_3$ at 125 MHz

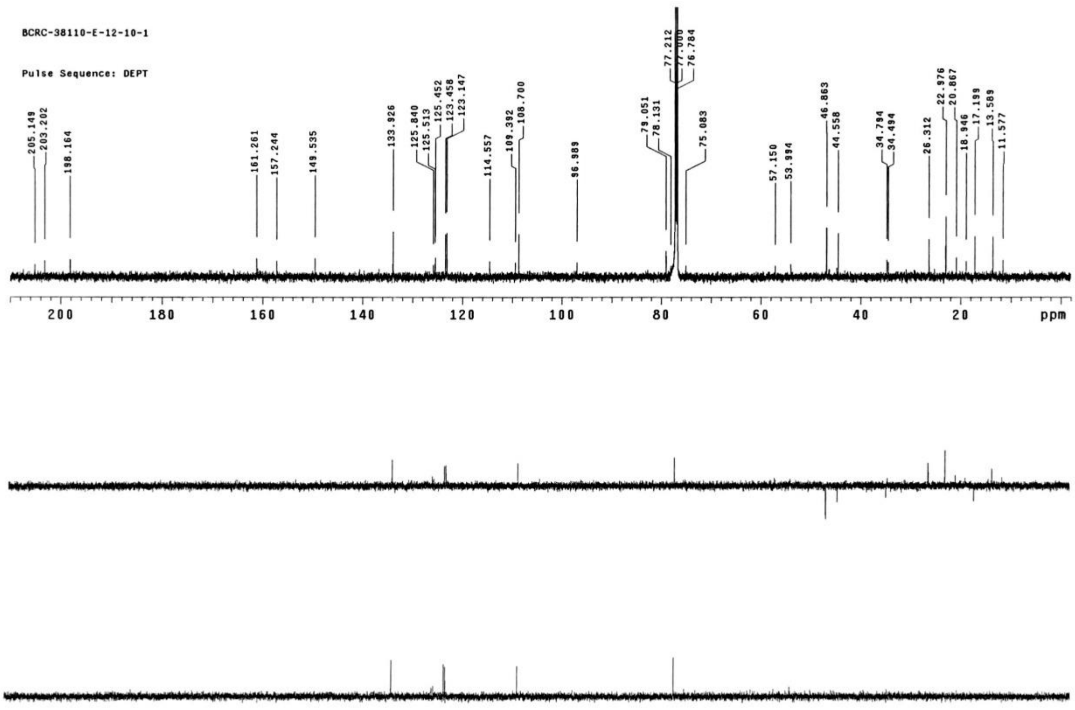

Figure S19. DEPT spectrum of monasxanthone A (**3**)

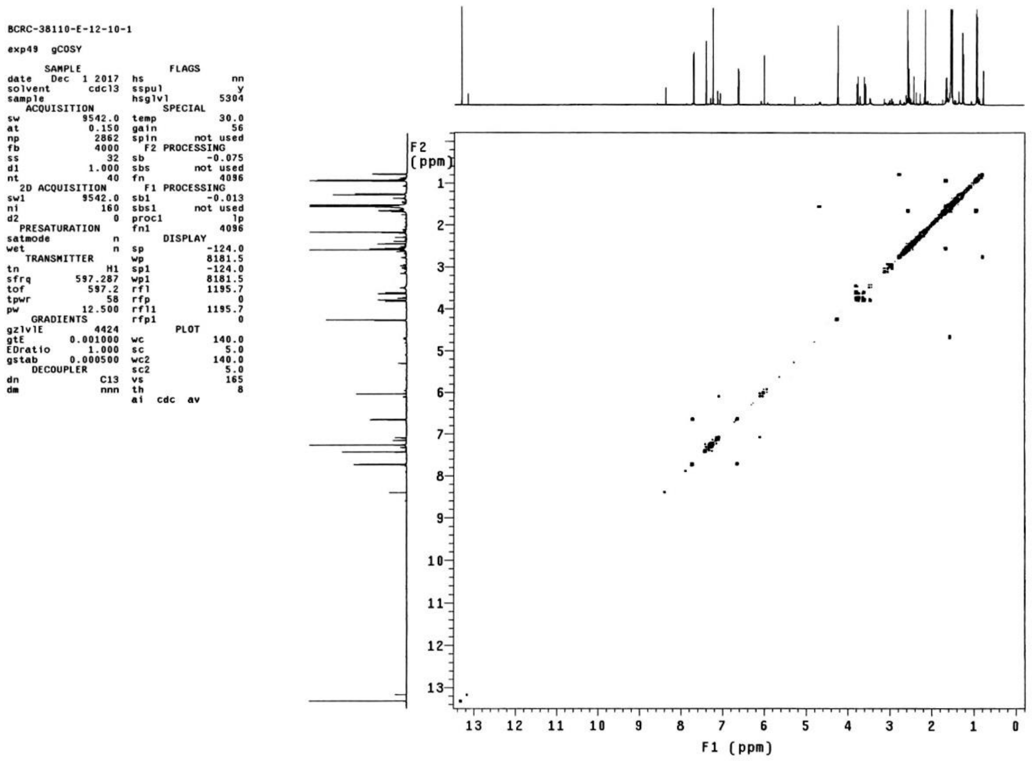

Figure S20. COSY spectrum of monasxanthone A (**3**)

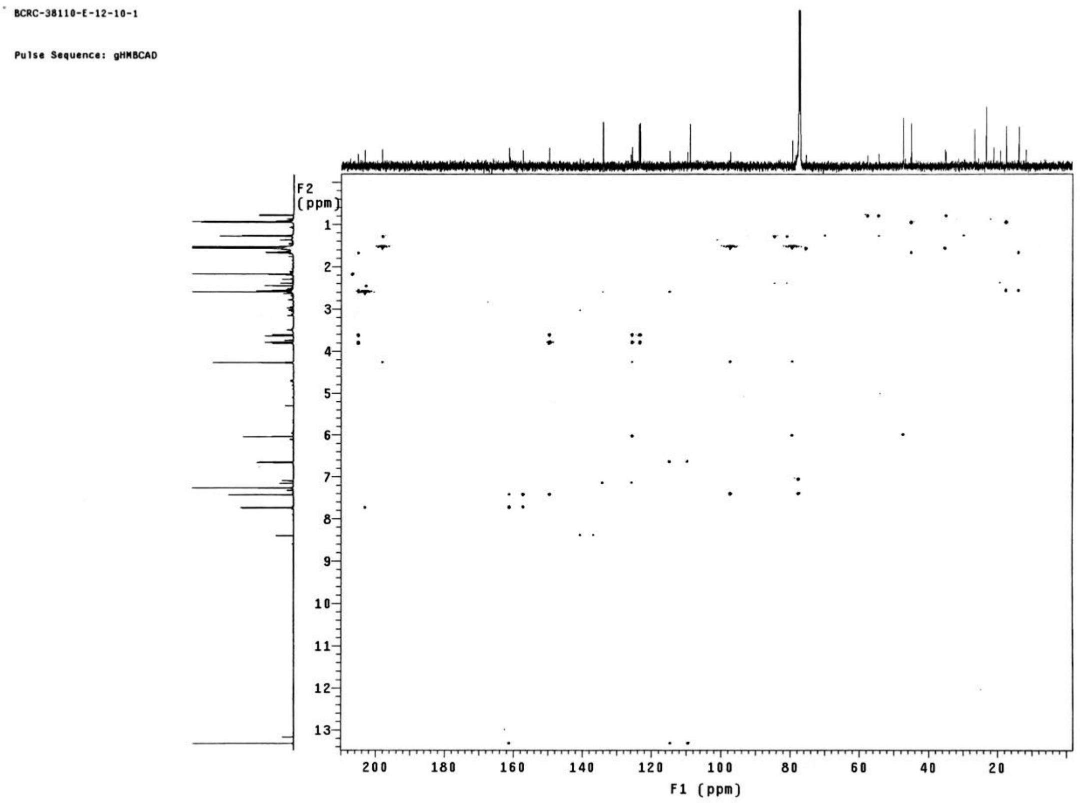

Figure S21. HMBC spectrum of monasxanthone A (**3**)

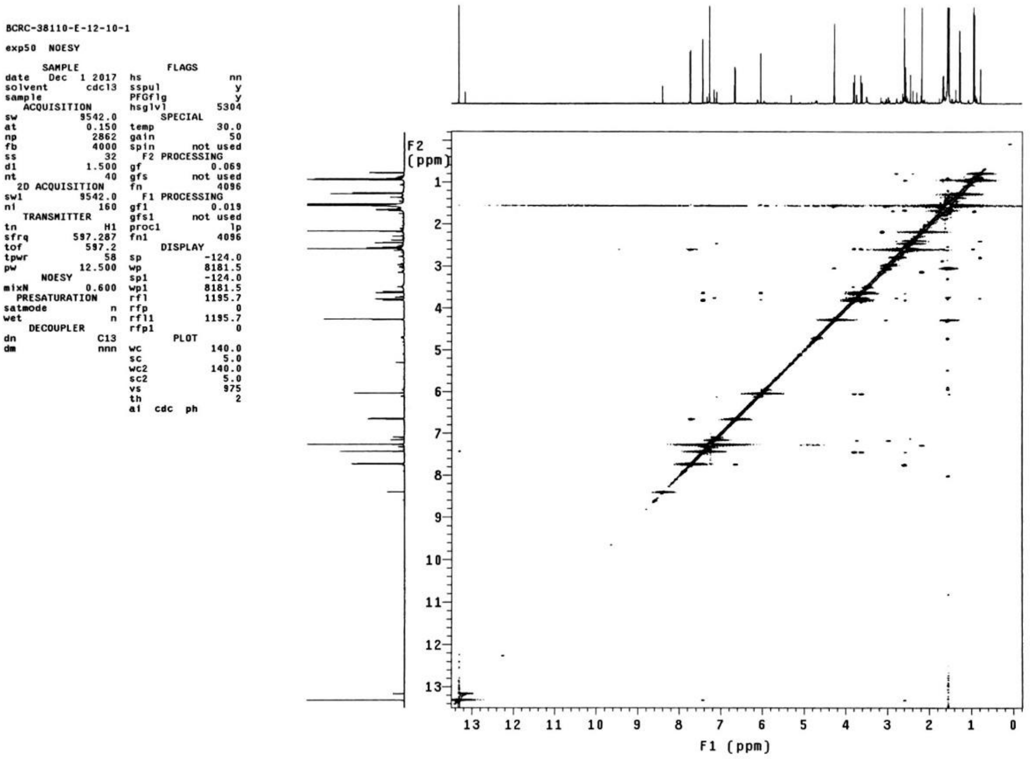

Figure S22. NOESY spectrum of monasxanthone A (**3**)

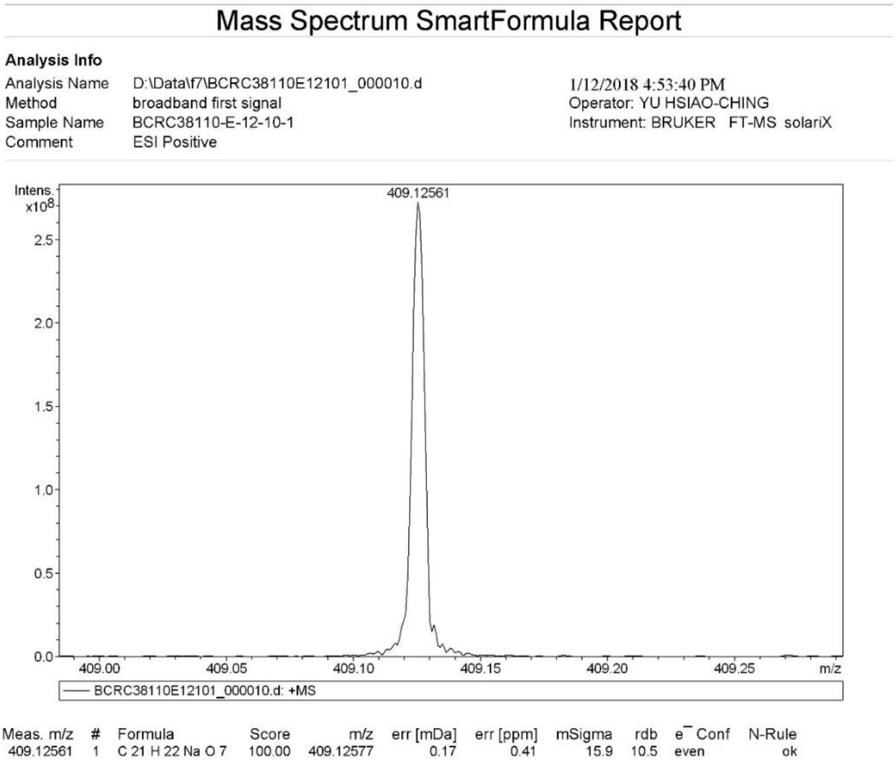

Figure S23. HRESIMS spectrum of monasxanthone A (**3**)

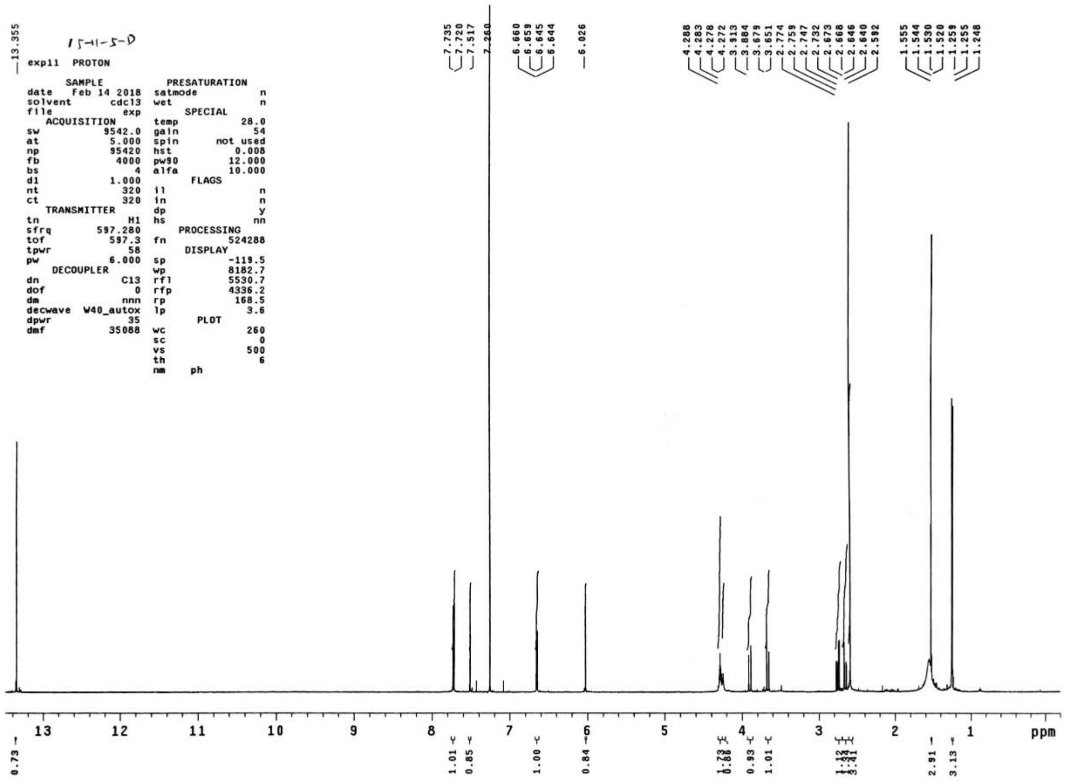

Figure S24. ^{1}H NMR spectrum of monasxanthone B (**4**) in $CDCl_3$ at 600 MHz

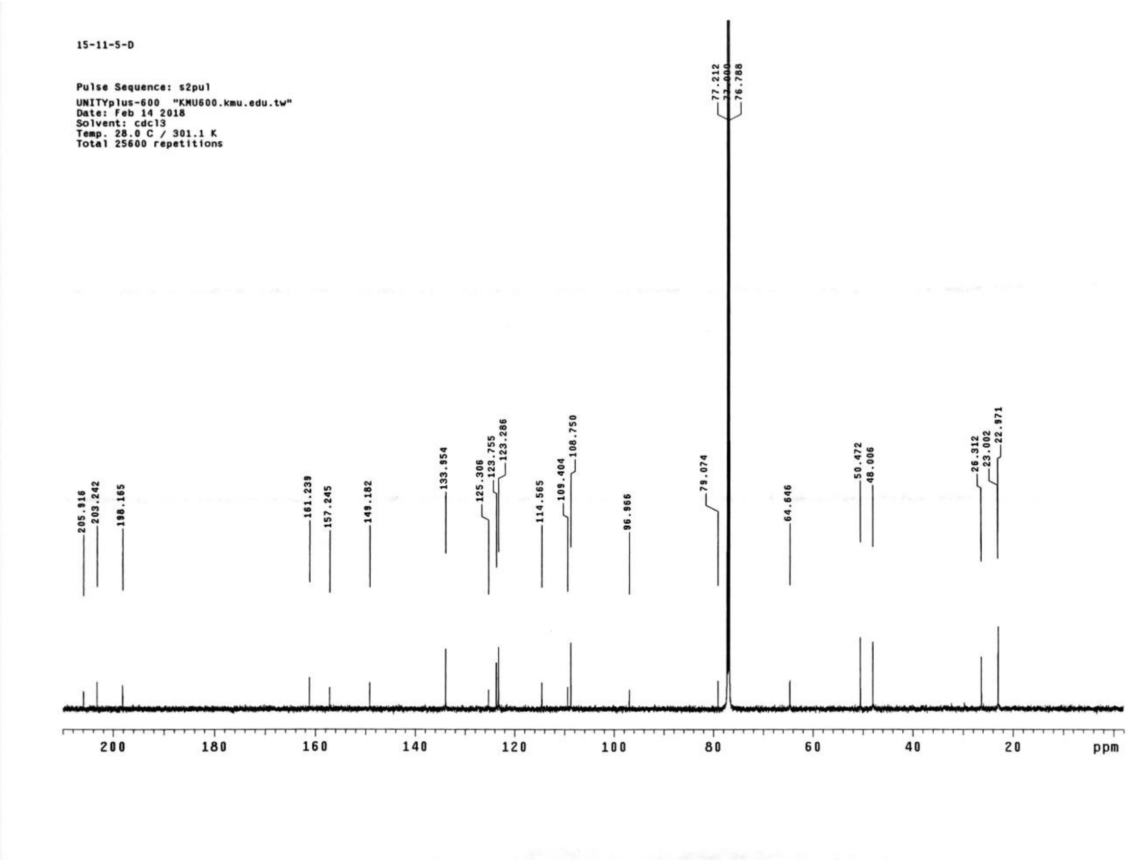

Figure S25. ^{13}C NMR spectrum of monasxanthone B (**4**) in $CDCl_3$ at 125 MHz

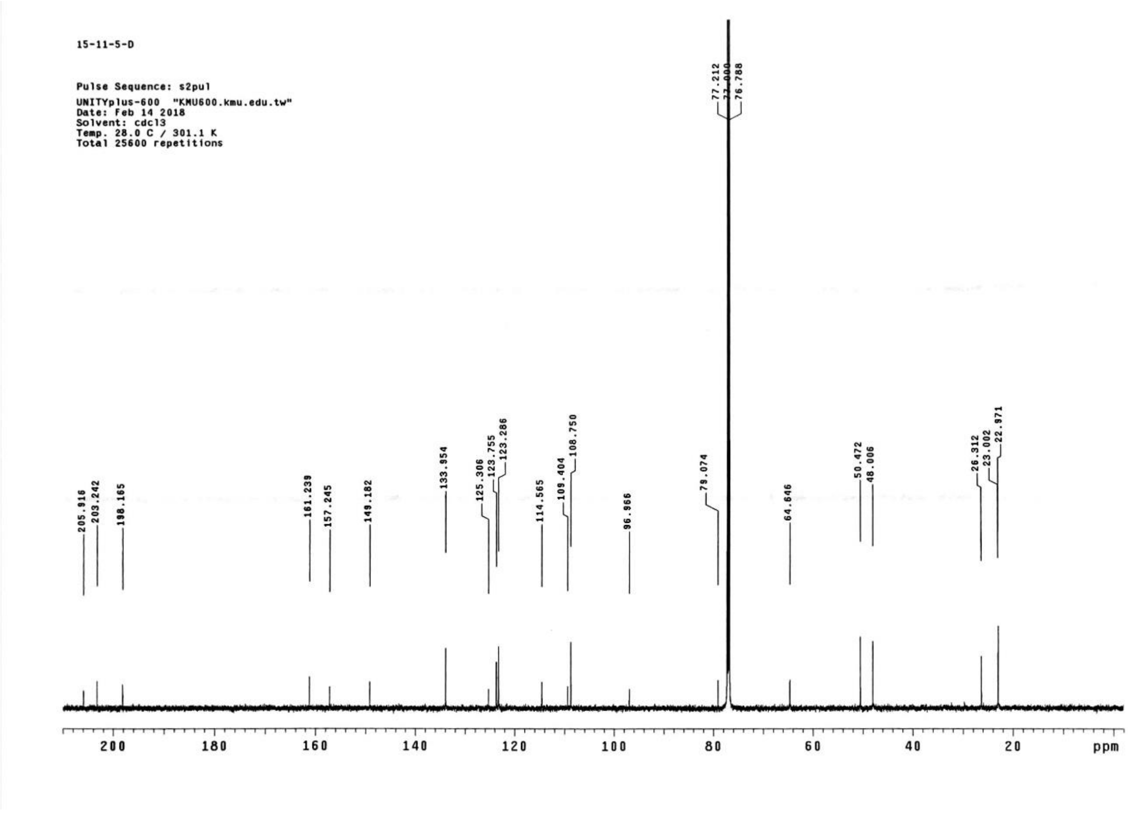

Figure S26. DEPT spectrum of monasxanthone B (**4**)

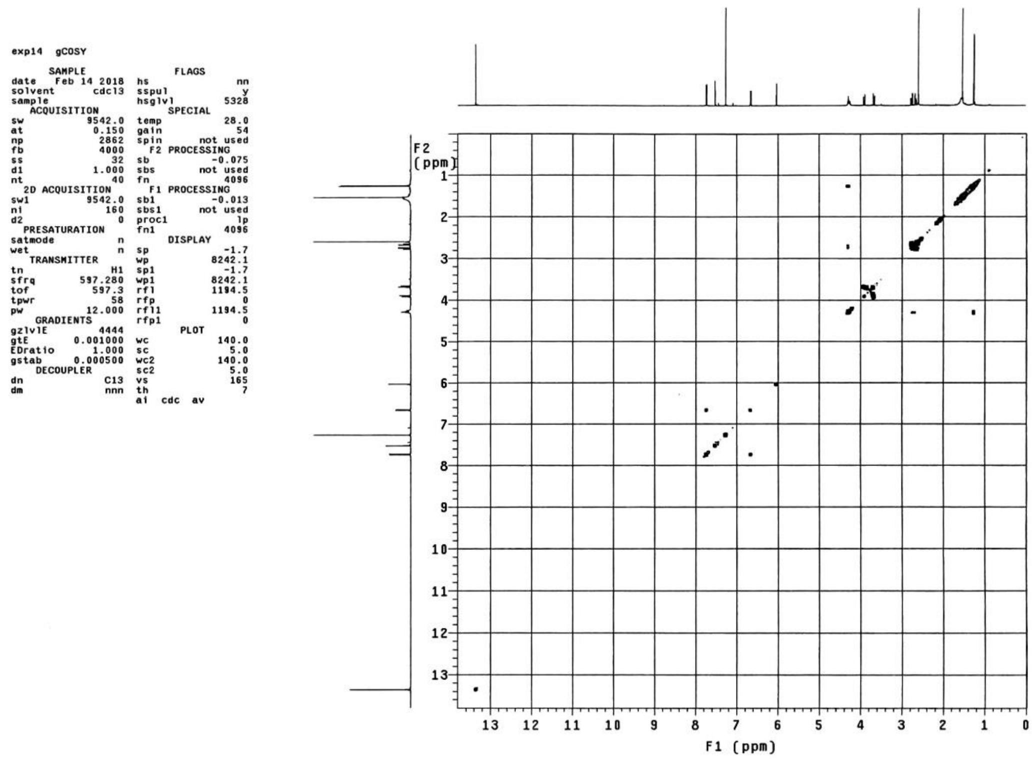

Figure S27. COSY spectrum of monasxanthone B (**4**)

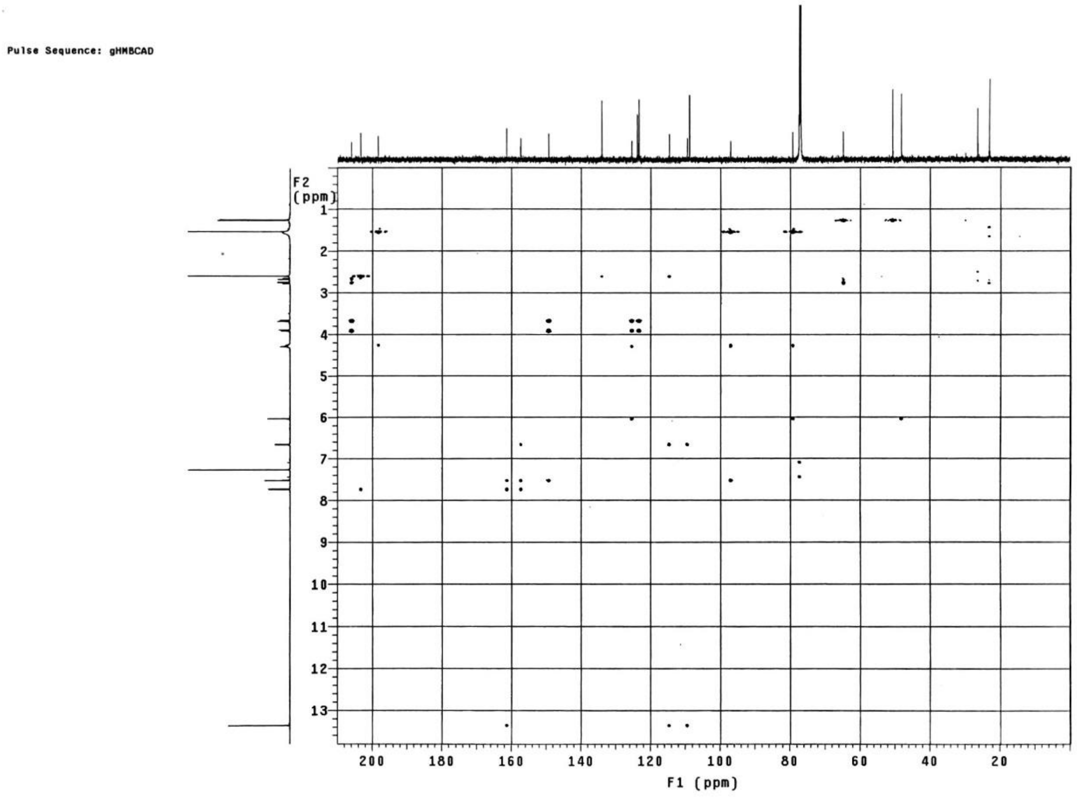

Figure S28. HMBC spectrum of monasxanthone B (**4**)

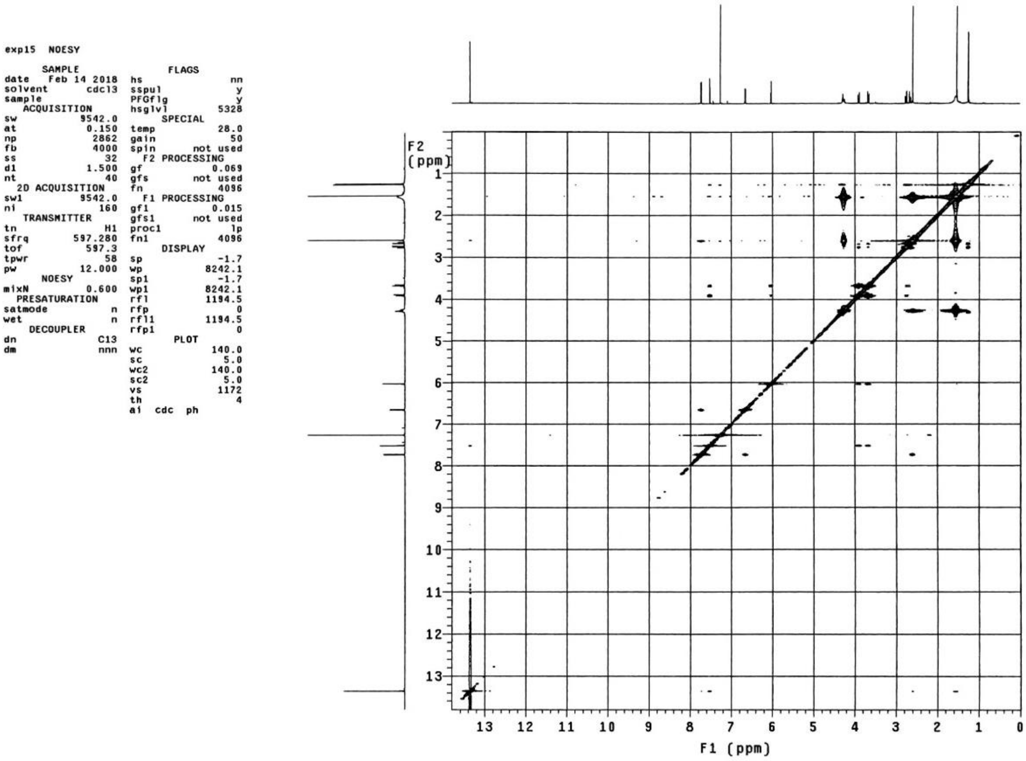

Figure S29. NOESY spectrum of monasxanthone B (**4**)

Mass Spectrum SmartFormula Report

Analysis Info

Analysis Name D:\Data\f7\BCRC38110E15115D_000002.d
Method broadband first signal
Sample Name BCRC48110-E-15-11-5-D
Comment ESI Positive

3/12/2018 2:54:25 PM
Operator: YU HSIAO-CHING
Instrument: BRUKER FT-MS solariX

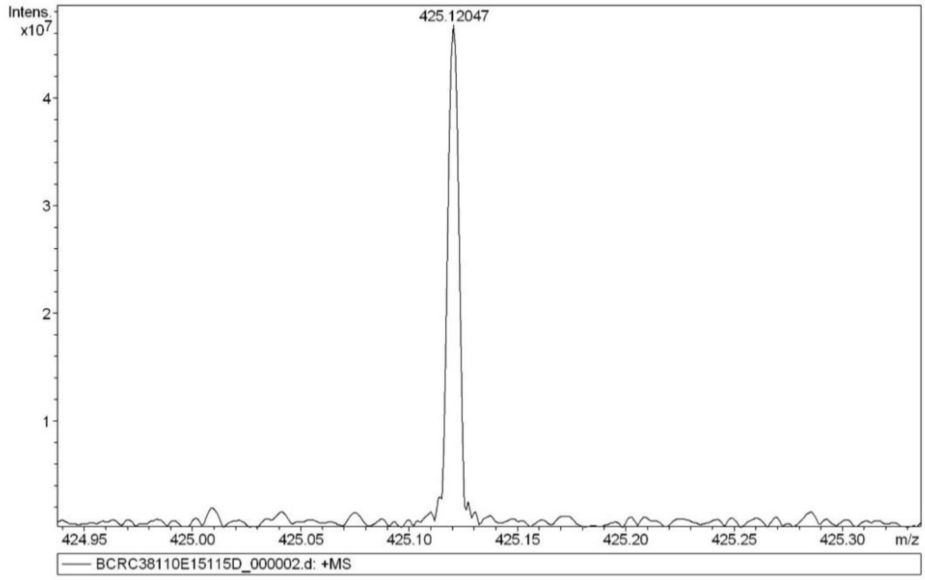

Meas. m/z	#	Formula	Score	m/z	err [mDa]	err [ppm]	mSigma	rdb	e⁻ Conf	N-Rule
425.12047	1	C 21 H 22 Na O 8	100.00	425.12069	0.22	0.52	20.6	10.5	even	ok

Figure S30. HRESIMS spectrum of monasxanthone B (**4**)

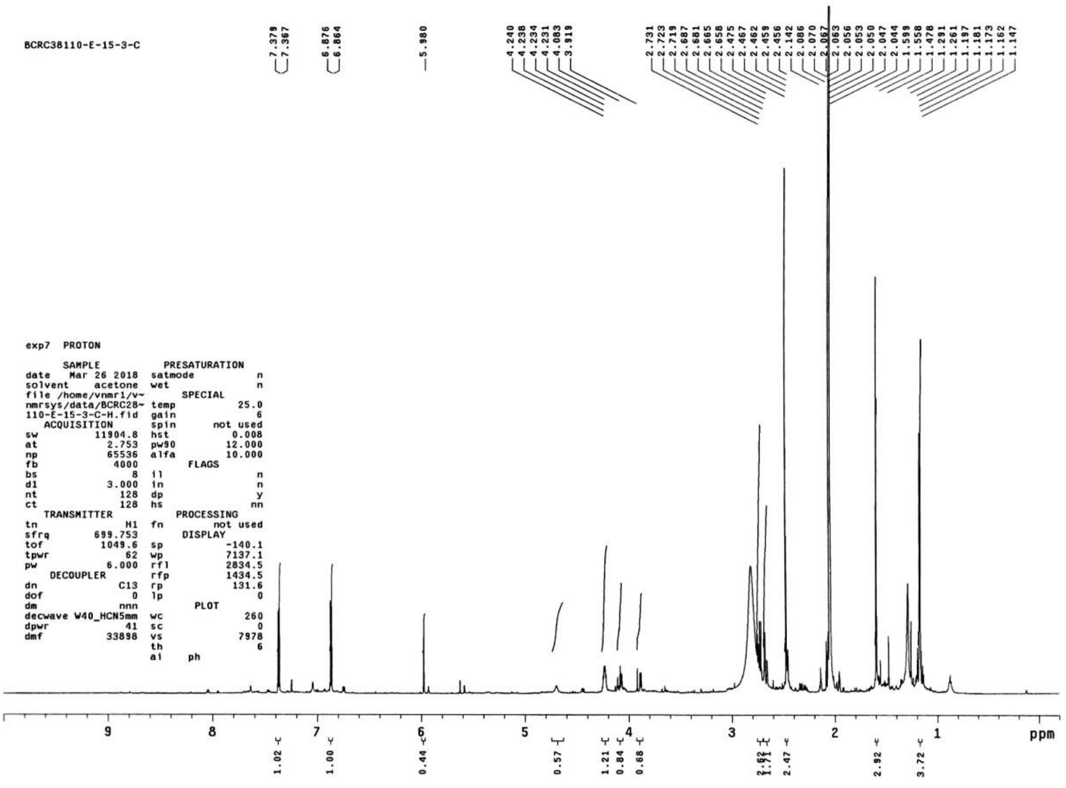

Figure S31. ^{1}H NMR spectrum of monasnaphthalenone (**5**) in acetone-d_6 at 600 MHz

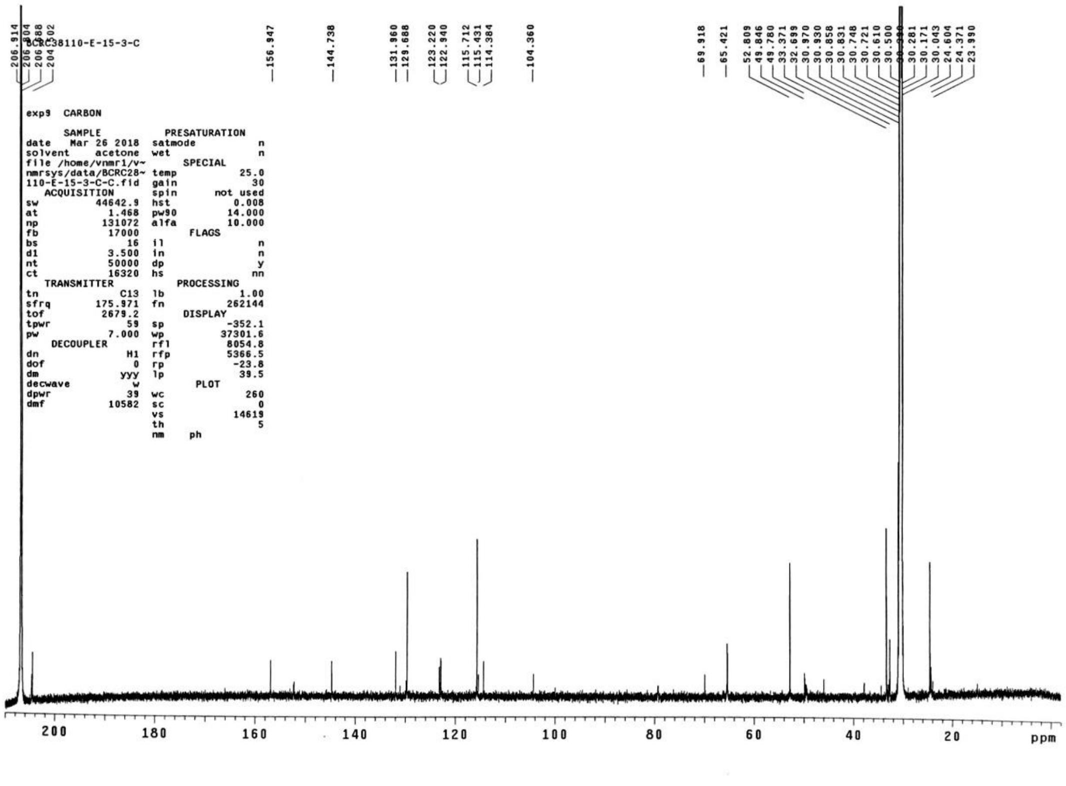

Figure S32. ^{13}C NMR spectrum of monasnaphthalenone (**5**) in acetone-d_6 at 125 MHz

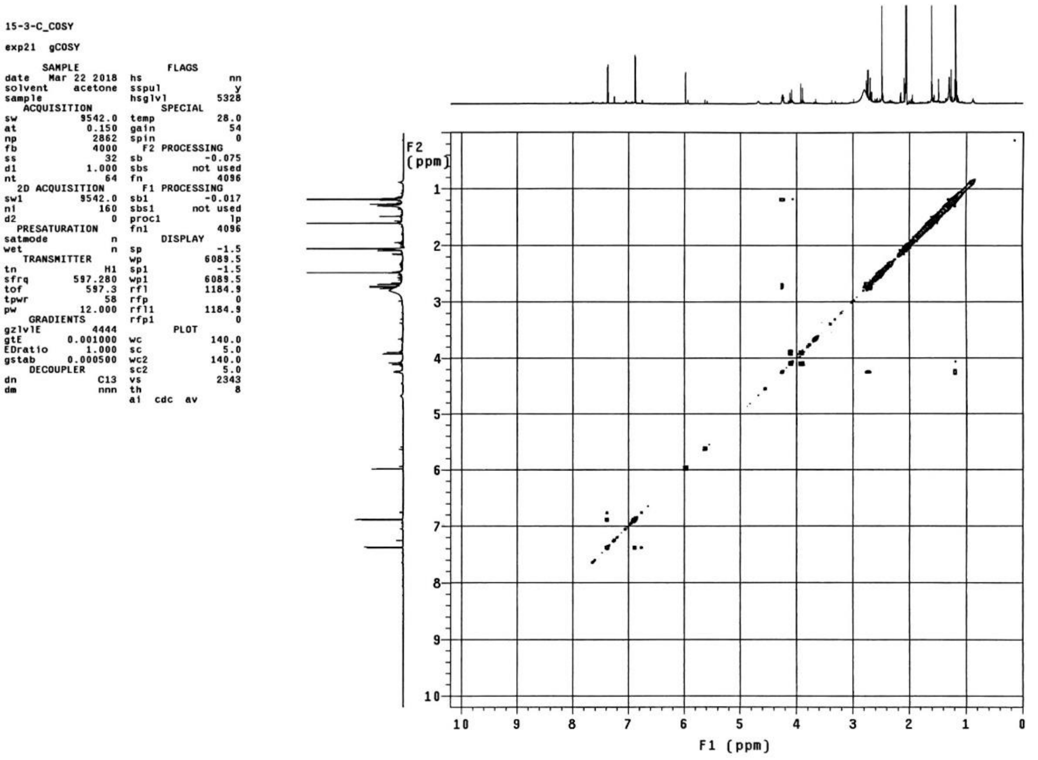

Figure S33. COSY spectrum of monasnaphthalenone (**5**)

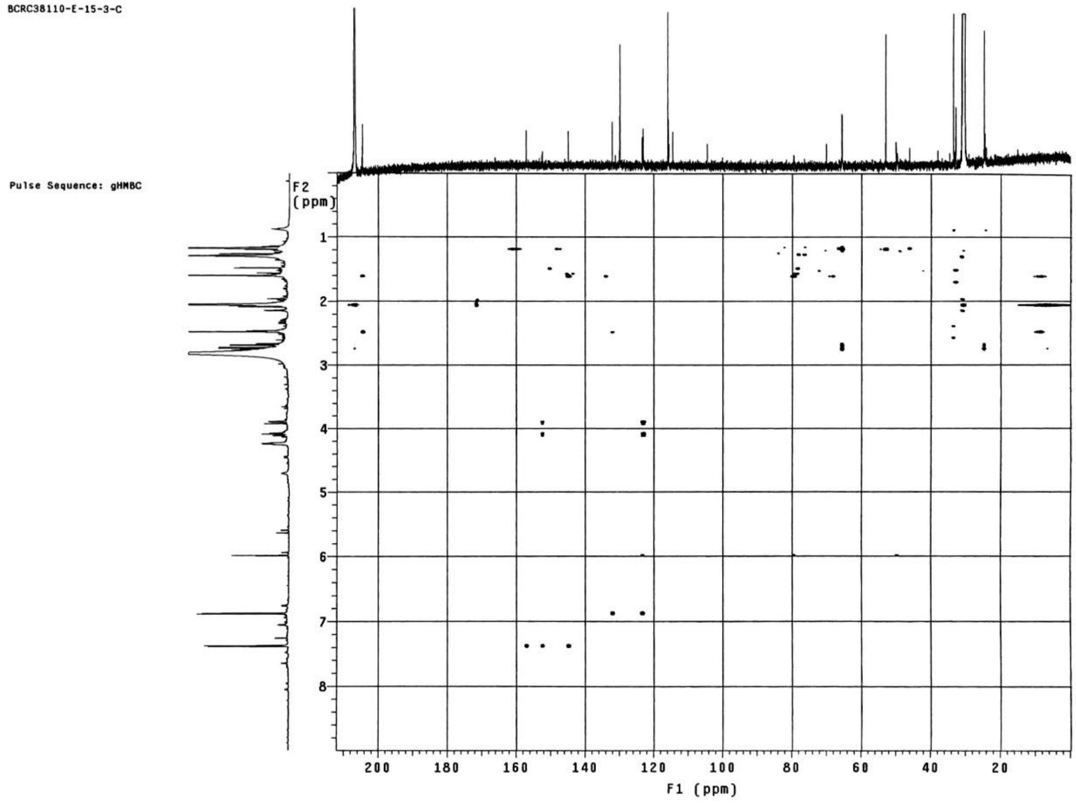

Figure S34. HMBC spectrum of monasnaphthalenone (**5**)

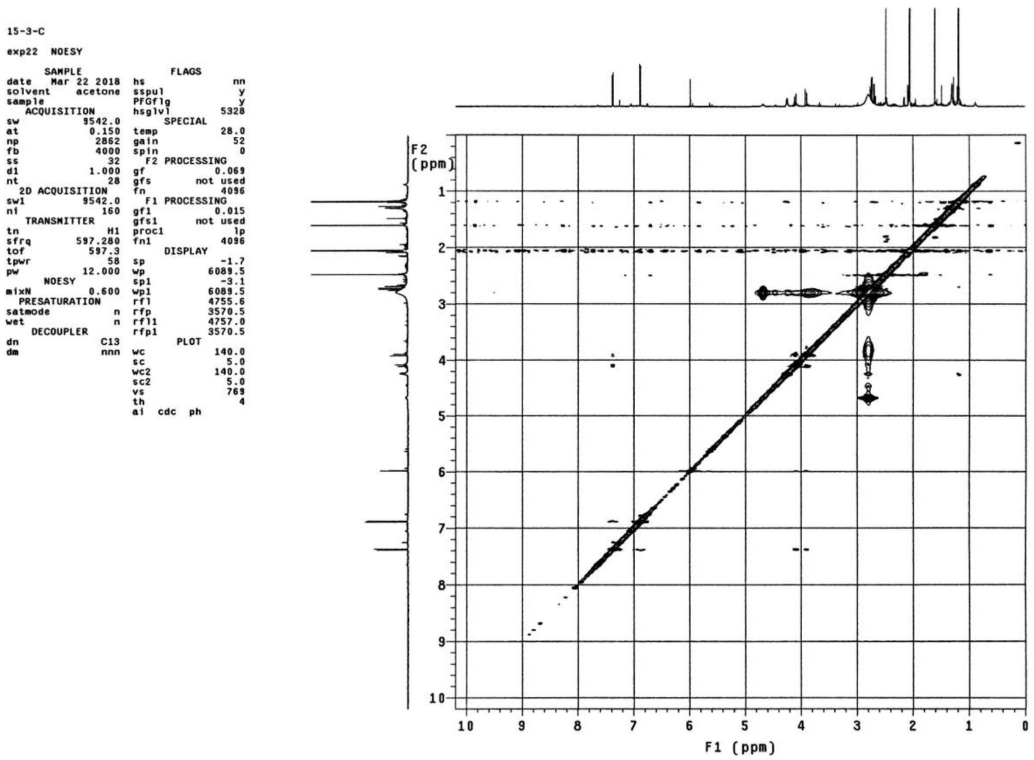

Figure S35. NOESY spectrum of monasnaphthalenone (**5**)

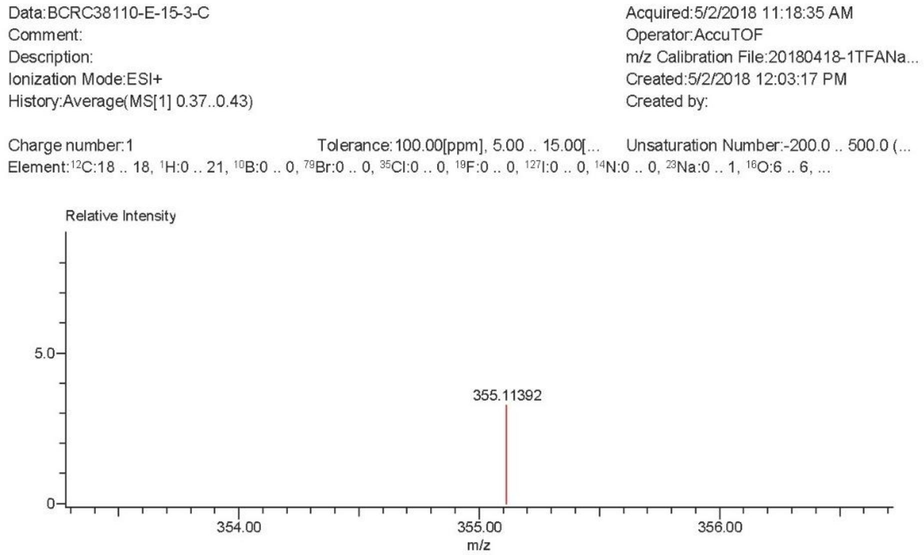

Mass	Intensity	Calc. Mass	Mass Difference [mDa]	Mass Difference [ppm]	Possible Formula
355.11392	1183.60	355.11576	-1.84	-5.17	$^{12}C_{18}{}^{1}H_{20}{}^{23}Na_{1}{}^{16}O_{6}$

Figure S36. HRESIMS spectrum of monasnaphthalenone (**5**)

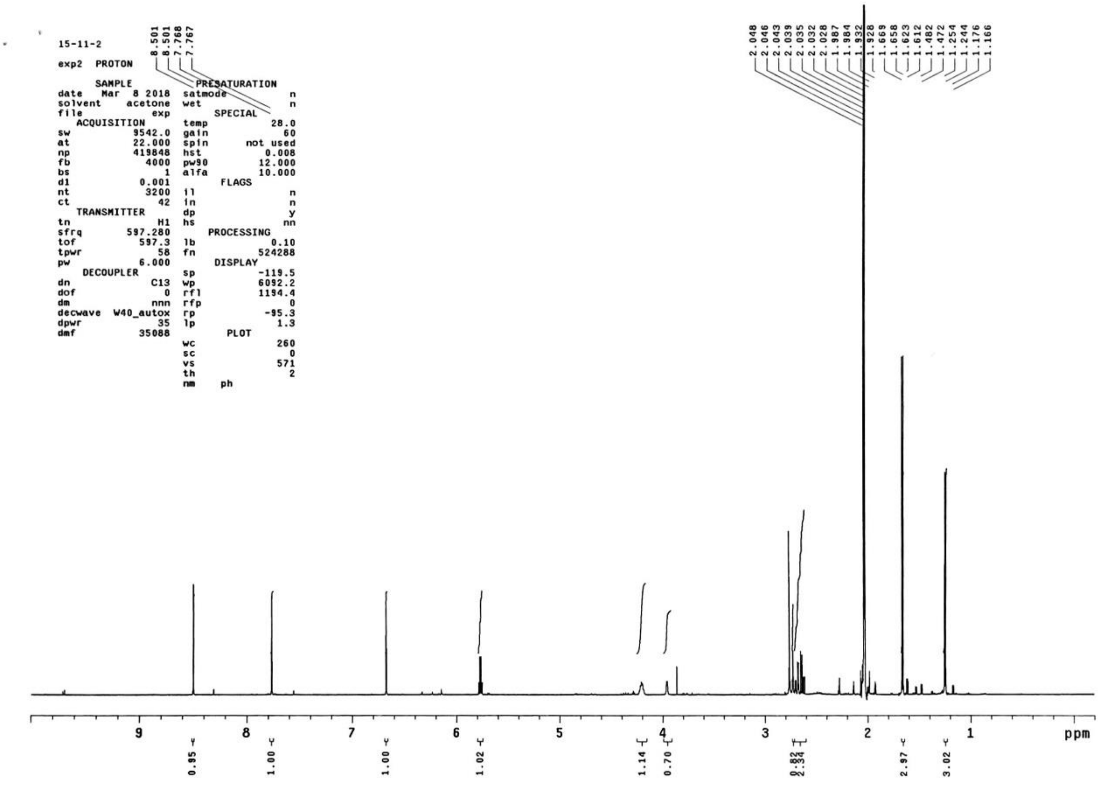

Figure S37. ^{1}H NMR spectrum of monapurpurin (**6**) in acetone-d_6 at 600 MHz

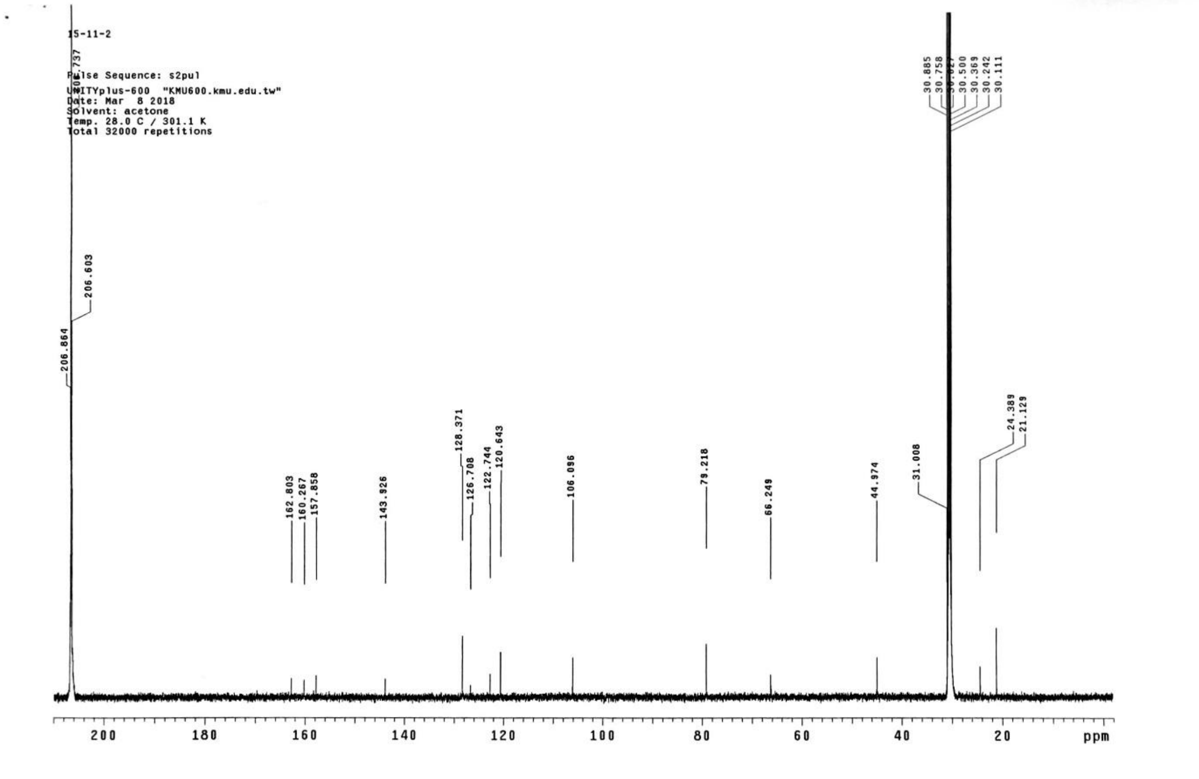

Figure S38. ^{13}C NMR spectrum of monapurpurin (**6**) in acetone-d_6 at 125 MHz

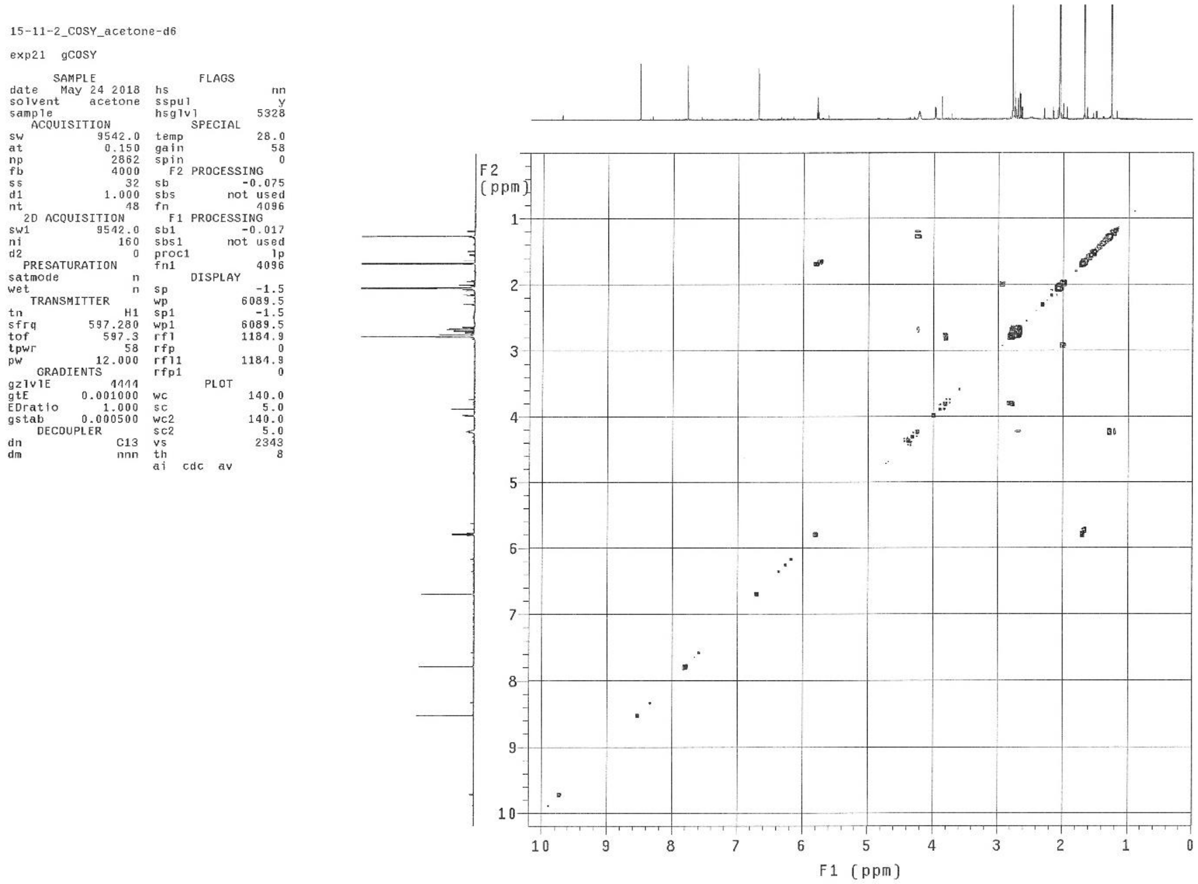

Figure S39. COSY spectrum of monapurpurin (**6**)

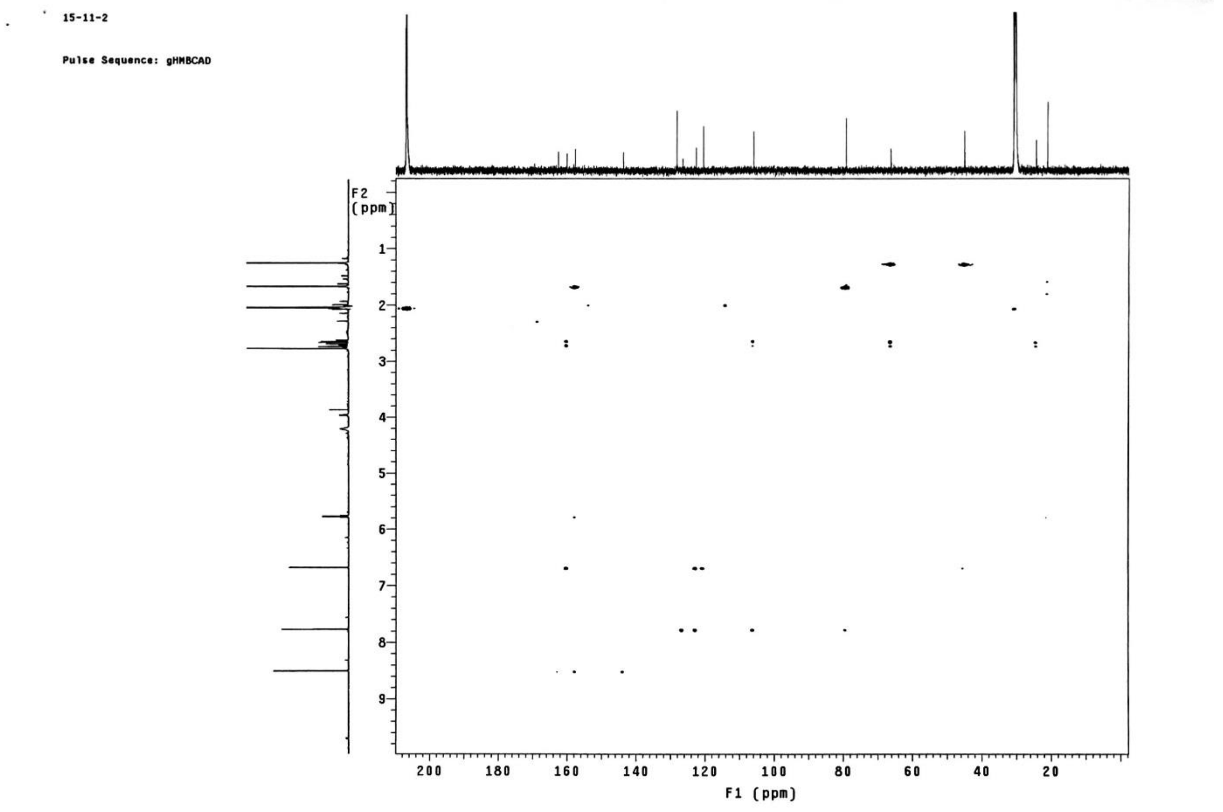

Figure S40. HMBC spectrum of monapurpurin (**6**)

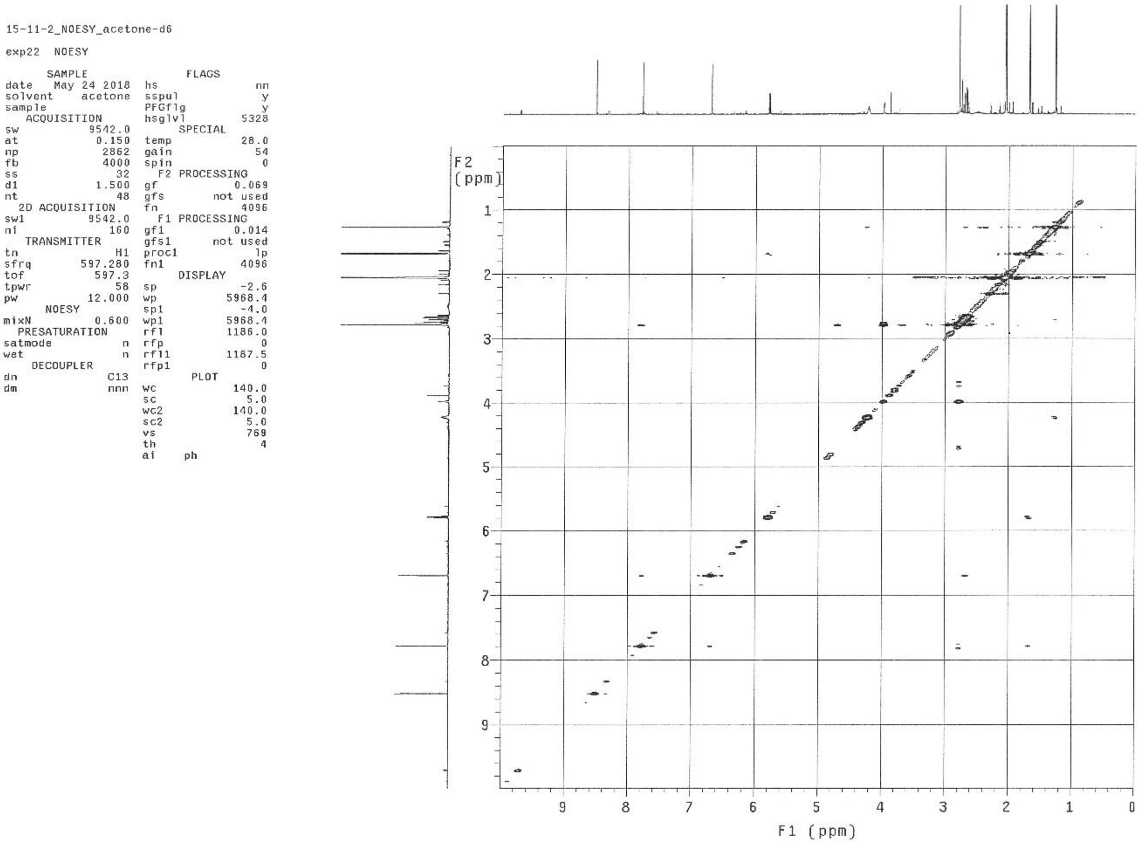

Figure S41. NOESY spectrum of monapurpurin (**6**)

Mass Spectrum SmartFormula Report

Analysis Info

Analysis Name	D:\Data\f7\BCRC38110E15112_000006.d	3/12/2018 3:04:04 PM
Method	broadband first signal	Operator: YU HSIAO-CHING
Sample Name	BCRC48110-E-15-11-2	Instrument: BRUKER FT-MS solariX
Comment	ESI Positive	

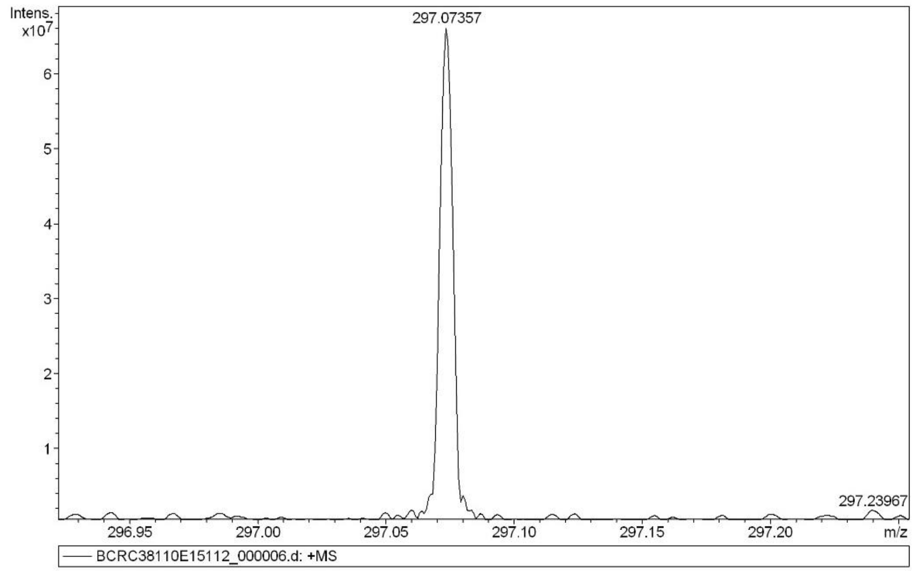

Meas. m/z	#	Formula	Score	m/z	err [mDa]	err [ppm]	mSigma	rdb	e⁻ Conf	N-Rule
297.07357	1	C 15 H 14 Na O 5	100.00	297.07334	-0.23	-0.76	16.0	8.5	even	ok

Figure S42. HRESIMS spectrum of monapurpurin (**6**)
